# Nano-in-Micro-Particles Consisting of PLGA Nanoparticles Embedded in Chitosan Microparticles via Spray-Drying Enhances Their Uptake in the Olfactory Mucosa

**DOI:** 10.3389/fphar.2021.732954

**Published:** 2021-09-01

**Authors:** Lena Marie Spindler, Andreas Feuerhake, Simone Ladel, Cemre Günday, Johannes Flamm, Nazende Günday-Türeli, Emre Türeli, Günter E. M. Tovar, Katharina Schindowski, Carmen Gruber-Traub

**Affiliations:** ^1^ Fraunhofer Institute for Interfacial Engineering and Biotechnology, Innovation Field Functional Surfaces and Materials, Fraunhofer-Gesellschaft, Stuttgart, Germany; ^2^ Institute of Interfacial Process Engineering and Plasma Technology, University of Stuttgart, Stuttgart, Germany; ^3^ Institute for Applied Biotechnology, Biberach University of Applied Science, Biberach, Germany; ^4^ Faculty of Natural Science, University of Ulm, Ulm, Germany; ^5^ MyBiotech GmbH, Überherrn, Germany

**Keywords:** intranasal application, mucosal uptake, poly lactic-co-glycolic acid particles, chitosan coating, nasal delivery, nano-in-micro particles, spray drying, olfactory transport mechanism

## Abstract

Intranasal delivery has gained prominence since 1990, when the olfactory mucosa was recognized as the window to the brain and the central nervous system (CNS); this has enabled the direct site specific targeting of neurological diseases for the first time. Intranasal delivery is a promising route because general limitations, such as the blood-brain barrier (BBB) are circumvented. In the treatment of multiple sclerosis (MS) or Alzheimer’s disease, for example, future treatment prospects include specialized particles as delivery vehicles. Poly(lactic-*co*-glycolic acid) (PLGA) nanoparticles are well known as promising delivery systems, especially in the area of nose-to-brain (N2B) delivery. Chitosan is also broadly known as a functional additive due to its ability to open tight junctions. In this study, we produced PLGA nanoparticles of different sizes and revealed for the first time their size-time-dependent uptake mechanism into the *lamina propria* of porcine olfactory mucosa. The intracellular uptake was observed for 80 and 175 nm within only 5 min after application to the epithelium. After 15 min, even 520 nm particles were detected, associated with nuclei. Especially the presence of only 520 nm particles in neuronal fibers is remarkable, implying transcellular and intracellular transport via the olfactory or the trigeminal nerve to the brain and the CNS. Additionally, we developed successfully specialized Nano-in-Micro particles (NiMPs) for the first time via spray drying, consisting of PLGA nanoparticles embedded into chitosan microparticles, characterized by high encapsulation efficiencies up to 51%, reproducible and uniform size distribution, as well as smooth surface. Application of NiMPs accelerated the uptake compared to purely applied PLGA nanoparticles. NiMPs were spread over the whole transverse section of the olfactory mucosa within 15 min. Faster uptake is attributed to additional paracellular transport, which was examined via tight-junction-opening. Furthermore, a separate chitosan penetration gradient of ∼150 µm caused by dissociation from PLGA nanoparticles was observed within 15 min in the *lamina propria*, which was demonstrated to be proportional to an immunoreactivity gradient of CD14. Due to the beneficial properties of the utilized chitosan-derivative, regarding molecular weight (150–300 kDa), degree of deacetylation (80%), and particle size (0.1–10 µm) we concluded that M2-macrophages herein initiated an anti-inflammatory reaction, which seems to already take place within 15 min following chitosan particle application. In conclusion, we demonstrated the possibility for PLGA nanoparticles, as well as for chitosan NiMPs, to take all three prominent intranasal delivery pathways to the brain and the CNS; namely transcellular, intracellular via neuronal cells, and paracellular transport.

## Introduction

Intranasal delivery became more prominent in the last decades because modern treatments focus on biopharmaceuticals and concurrently reveal opportunities, but also provide challenges. Intranasal delivery is a promising route to overcome general limitations such as the blood-brain barrier (BBB). In the treatment of neurological disorders, for example multiple sclerosis or Alzheimer’s disease. Future prospective treatments may also include not only proteins or antibodies, but also specialized particles as delivery vehicles, but the BBB is shielding the central nervous system (CNS) and the brain from the blood stream. Modern therapeutics, such as antibodies and proteins, are therefore banned through size exclusion and surface charge (Wolburg et al., 1994; Stützle et al., 2015), which is problematic for intravenous and oral administration in current therapeutic approaches. More than one billion people are currently affected by neurological diseases with a rising tendency (WHO 2006). Further, the global need displays a great demand for effective treatments of the upper airways, such as the nasal cavity. Covid-19 infections, as one prominent example, occur mainly from the initial contact to the nasal mucosa and can harm the brain (Yu et al., 2020; Layden et al., 2020). Therefore, to prevent greater damage, effective treatments should also occur along this route. As a consequence, the actual need for innovative intranasal delivery systems is rapidly increasing.

The nasal mucosa, as the first biological barrier in the nose to target the brain and the CNS, consists of respiratory and olfactory mucosa. The respiratory epithelium covers approximately 97% of the nasal cavity in humans and is necessary for respiration (Ugwoke et al., 2005). Further, rapid clearance of dust and other foreign substances occurs via the directed movement of millions of cilia (Menache et al., 1997). In contrast to this, the olfactory mucosa is characterized by fewer long cilia, specialized to detect odorants, initializing an electric signal in olfactory sensory neurons to maintain olfaction (Ugwoke et al., 2005). The olfactory sensory neurons are located between epithelial cells and underlying basal cells, forming neuronal bundles, which protrude through the ethmoid bone and therefore directly connect the olfactory mucosa to the brain and the CNS (Morrison and Costanzo 1990; Morrison and Costanzo 1992). Further, the olfactory mucosa consists of a *lamina propria* mainly built of fibroblastic cells. The olfactory epithelium covers 4–5 cm^2^ in humans. Up to approximately 10 cm^2^ of the olfactory mucosa can be excised from the dorsal part of the *concha nasalis dorsalis* in pigs (Getty 1975; Mistry 2009; Ladel et al., 2018). The olfactory epithelium can be distinguished from the respiratory epithelium due to fewer blood capillaries and hence brighter color (Mistry 2009; Stützle et al., 2015).

Polymeric nanoparticles embody convincing advantages such as stability, high loading capacity, and controlled drug release (Bourganis et al., 2018; Rabiee et al., 2020). Poly(lactic-*co*-glycolic acid) (PLGA) nanoparticles especially are widely known as promising delivery vehicles. PLGA, consisting of lactic and glycolic acid, is a biodegradable biopolymer already approved by the Food and Drug Administration (FDA) and the European Medicine Agency (EMA) (Danhier et al., 2012). Its release properties are tunable due to the mass ratio of lactic and glycolic acid. Exemplarily, a mass ratio of 50% each results in a controlled release over several days. Degradation occurs through hydrolysis and the decomposition products are further metabolized. U. Seju et al. (2011) reported PLGA nanoparticles as promising delivery systems for nose-to-brain delivery because drug release of olanzapine occurs over several days due to slow swelling of PLGA and long-term residence of the resulting gel at the site of action, further 8-fold drug concentrations, compared to the solute form, have been achieved (Seju et al., 2011). Muntimadugu et al. (2016) reported the transport of PLGA nanoparticles with diameters below 200 nm along axons within the nasal mucosa (Muntimadugu et al., 2016). Neuronal bundles within the olfactory mucosa being directly connected to the olfactory or trigeminal nerve is identified as one transport route to the brain and the CNS (Anderson et al., 2008; Stützle et al., 2015; Gänger and Schindowski 2018). PLGA and chitosan particles up to 250 µm were recently reported to be mainly transported via the olfactory route (Rabiee et al., 2020); however, uptake and distribution examinations of PLGA and chitosan particles in nasal mucosa samples are still rare.

Additional nose-to-brain delivery routes are intracellular uptake and further transcytosis, as well as paracellular transport. Other research groups focused on the paracellular route in epithelial cell cultures and mucosal tissues (Sadeghi et al., 2008; Fazil et al., 2012; Sonaje et al., 2012; Rassu et al., 2016). Herein chitosan-based carriers have become one of the most promising and intensively-studied mucosal drug delivery systems (Garcia-Fuentes and Alonso 2012). This is attributed to their mild and simple preparation technique as well as their capacity to associate biologics and enable transport across mucosal barriers. Chitosan is a renewable biopolymer, which is gained from the naturally occurring chitin of crustaceans, extracted by deacetylation (Wenling et al., 2005; Yuan et al., 2011; Sarmento and Neves 2012). With at least 50% N-deacetylated chemical groups, it is referred to as chitosan. The origin, as well as the degree of deacetylation, affects the polymer properties, such as solubility, swelling, and adhesion. The interaction between the positive amino groups of chitosan and several negative groups in the mucosa enables mucoadhesion, which is further dependent on the molecular weight of the chitosan (Henriksen et al., 1996). The length of the chitosan polymer chains and their average molecular weight can be controlled by enzyme-mediated decomposition or acid hydrolysis (Ilium 1998). The chitosan utilized in this study, for example, has 80% deacetylated residues and is; therefore, mucoadhesive and well soluble in acidic solutions. Chitosan can open tight junctions (Fazil et al., 2012; Sonaje et al., 2012; Rassu et al., 2016), which are the characteristic intercellular connections present in epithelial cells and endothelial cells. One major component *Zonula occludens* protein 1 (*ZO-1*) was initially explored, in 1986, by Stevenson et al. (1986) in epithelial cells (Stevenson et al., 1986), it was later also documented in endothelial cells (Anderson et al., 1988) and fibroblasts (Itoh et al., 1991; Howarth et al., 1992). The above-mentioned research groups described the ability of chitosan to open tight junctions in cell culture, studying the impact of chitosan solutions on cellular interconnections. Further, other research groups have postulated that chitosan nanoparticles open tight junctions and; therewith, affect increased drug levels in the brain and the CNS, but did not directly prove the tight junction opening (Rassu et al., 2016; Bourganis et al., 2018; Rabiee et al., 2020). Further experimental investigations of tight junction opening are, therefore, still needed to fully clarify intranasal transport routes of chitosan particles and encapsulated active pharmaceutical ingredients.

Together with the application of particles to the nasal mucosa, it is important to consider particle distribution in the olfactory tissue, biocompatibility, biodegradation, and especially immune response. Nevertheless, these aspects are rarely reported in literature for intranasal delivery and more research is needed (Rabiee et al., 2020). The nasal mucosa, as a biological barrier directly exposed to the environment, is a well-known immune active tissue (Morrison and Costanzo 1990; Doty, 2015; Stützle et al., 2015; Bourganis et al., 2018; Ladel et al., 2018). Nasal-associated lymphoid tissue (NALT) includes immune active cells, either present between the epithelial layer and the *lamina propria*, infiltrated from blood vessels, or directly produced inside of lymphoid follicles located in the mucosa itself. Moreover, recent studies have revealed the still incomplete understanding of intranasal pathways and distribution routes of individual compounds, as well as formulation compositions (Rabiee et al., 2020; Keller et al., 2021). Which drug delivery system exactly triggers what specific pathway is mainly unclear up to now. Consequently, clarification of distribution routes of individual compounds as well as formulation composition, such as the herein investigated prominent particle materials PLGA and chitosan, are needed to develop suitable drug delivery systems in the future.

Within this study, we examined size- and time- dependent particle permeation studies on porcine olfactory mucosa. We compared the permeation of pure PLGA nanoparticles of different diameters up to 520 nm to spray-dried nano-in-micro particles (NiMPs) coated with chitosan to identify chitosan-mediated mechanisms important for intranasal delivery. Additionally, we utilized the unique benefits of the controlled release of PLGA nanoparticles over several days combined with the aforementioned abilities of chitosan. Moreover, we investigated organoid accumulation, such as in glands and neuronal bundles as well as the local initial immune response within the olfactory mucosa.

## Materials and Methods

### Nanoparticle Preparation

Poly(lactic-*co*-glycolic acid) PLGA nanoparticles with 80 and 175 nm diameter were prepared by precipitation. 5 mg ml^−1^ PLGA 50:50 (Evonik, Essen, Germany) and 14 μg ml^−1^ Lumogen (BTC Europe, Monheim, Germany), or 10 mg ml^−1^ PLGA 50:50 (Evonik, Essen, Germany) and 28 μg ml^−1^ Lumogen (BTC Europe, Monheim, Germany) were dissolved in Acetone for 80 and 175 nm particles respectively. This solution was mixed in a 1:2 solvent:nonsolvent ratio with distilled water containing 2.5 mg ml^−1^ Pluronic F68 (Sigma-Aldrich, St. Louis, United States) by the use of a magnetic stirrer.

PLGA nanoparticles with 520 nm diameter were prepared by two-step double emulsification to obtain water-oil-water emulsions (w/o/w). The first emulsification step was performed with Milli-Q water and 5 mg ml^−1^ PLGA (Evonik, Essen, Germany) solution in Dichloromethane:Ethylacetate (1:3) as solvent. The water to organic phase ratio was 1:10. For labeling 28 μg ml^−1^ Lumogen F Red (BTC Europe, Monheim, Germany) was added to the formulation dissolved in the organic phase. As a surfactant polyvinyl alcohol (PVA) with a molecular weight of 27 kDa (Sigma-Aldrich, St. Louis, United States) was used in the second emulsification. Therefore 1 %wt. PVA was dissolved in water at 80°C. The obtained primary emulsion and PVA containing aqueous phase were emulsified to achieve a w/o/w emulsion at a ratio of 1:10. Both emulsification steps were performed at the highest speed (26,000 rpm) for 1 min by using Silence Crusher M (Heidolph Instruments GmbH & Co. KG, Schwabach, Germany) homogenization device. The finally obtained w/o/w emulsion was stirred moderately overnight to evaporate the organic phase.

### Nano-in-Micro Particle Preparation via Spray Drying

A Büchi B-290 mini spray dryer (Büchi Labortechnik AG, Flawil, Switzerland) was used to encapsulate the previously prepared PLGA nanoparticles into a chitosan matrix. Chitosan 80/200 (Chitoceuticals, Heppe Medical Chitosan GmbH, Halle, Germany), with a degree of deacetylation of 80% and a molecular weight of 150–300 kDa, was dissolved in 0.5 %vol. acetic acid at a concentration of 1 %wt. The chitosan matrix was labeled with fluorescein sodium salt (Honeywell Flunka, New Jersey, United States) at a concentration of 260 μg ml^−1^ in the spray drying solution. The nanoparticle suspension (6.25 mg ml^−1^ PLGA in Milli-Q water) was then mixed with the fluorescein labelled chitosan solution at a mass ratio of 1/3 or 2/3 before spray drying. Spray drying was performed with a two-fluid nozzle and a nozzle cap diameter of 1.5 mm. The inlet temperature was set to 100°C at a fluid feed rate of 4.46 ml min^−1^, gas feed rate of 12.94 g min^−1^, and an aspiration rate of 100% equating with ∼700 g min^−1^. The system was equilibrated before use and washed after spray drying with Milli-Q water.

### Scanning Electron Microscopy

For Scanning Electron Microscopy (SEM) analysis, PLGA nanoparticle suspension (6.25 mg ml^−1^ PLGA in Milli-Q water) was vortexed and 10 µl of the suspension was pipetted onto a clean silicon wafer, covered with a petri dish, and dried overnight at room temperature. Nano-in-micro particle powders were fixed with conductive electron microscopy tape directly onto a sample holder. A thin layer of platinum was sputtered on all samples before measurement. Measurements were performed at different magnifications with a Leo Gemini 1530 VP (Carl Zeiss Microscopy Deutschland GmbH, Oberkochen, Germany) microscope at 5.00 kV using the InLens detector and SE2 for signal B.

### Laser Scanning Microscopy

For Confocal Laser Scanning Microscopy (CLSM) the nanoparticle suspension (6.25 mg ml^−1^ PLGA in Milli-Q water) was vortexed and 10 µl were applied to a cover glass, which was then covered with a petri dish and dried at room temperature protected from light. Completely dried samples were measured upside down with a 63x water immersion objective. Nano-in-micro particles were deposited rarely with a sieve and a spatula onto a cover glass, covered with an additional cover glass and sealed gently with tape, to avoid contamination during analysis. Powder samples were measured immediately after preparation with the 63x water immersion objective. Most tissue samples have been measured with a 20x objective. All measurements have been performed with a confocal LSM 710 (Carl Zeiss Microscopy Deutschland GmbH, Oberkochen, Germany). The utilized lasers and the corresponding emission filters for CLSM imaging are displayed in Supplementary Table S1. For three-dimensional information, z-stack measurements with a pitch of 0.5 µm for 63x recordings and 1 µm for 20x images were recorded and stitched together in a maximum intensity projection. For picture analysis and post-processing, ZEN software (Carl Zeiss Microscopy Deutschland GmbH, Oberkochen, Germany) was used.

### Particle Size Distribution

PLGA nanoparticles were measured as a suspension in Milli-Q water via dynamic light scattering using a Zetasizer Nano ZS (Malvern Instruments, Malvern, United Kingdom). Mean particle size and Polydispersity Index (PI), referred to as squared difference between standard deviation and average decay rate, were reported (ISO 2017). The average decay rate is defined by diffusion coefficient and scattering vector as written in ISO standard 22412:2017. Since the Stokes-Einstein hydrodynamic diameter is inversely proportional to the decay rate, the average diameter is equal to the average decay rate.

Nano-in-micro particles were measured via static light scattering at 1,500 rpm stirring velocity performed with a Mastersizer 2000 (Malvern Instruments, Malvern, United Kingdom) using its µP 2000 measurement cell. The spray-dried powder sample was first dispersed in 2-propanol via ultrasonic treatment for 3 s in an ultrasonic bath. Additional measurements at 1,500 rpm were performed after 1 min ultrasonic treatment in the measurement cell. Measuring chitosan particles is reported with the organic solvent ethanol (Kašpar et al., 2013; Tokárová et al., 2013), but we detected swelling of chitosan particles within several minutes in ethanol falsifying the particle size measurement as a result. Therefore, we only used 2-propanol as a solvent for static light scattering measurements. We calculated the mean particle diameter from the volume distribution output. Additionally, Scanning Electron Microscopy (SEM) images were analyzed with ImageJ software (Wayne Rasband, 1997). Particle diameters were extracted, plotted, and fitted with a Gaussian fit in Origin Pro 2019 (Origin Lab, 1992).

Further, the Polydispersity Index (PdI) also for nano-in-micro particles (NiMPs) was calculated with Eq. 1 using the mean particle diameter $d_{m}$ and the standard deviation of the particle size σ (Clayton et al., 2016), which is not automatically reported within the Mastersizer software (Malvern Instruments, Malvern, United Kingdom).

$$PdI={\left(\frac{\sigma }{d_{m}}\right)}^{2}$$ (1)

### Zeta Potential

Zeta potential was examined for PLGA nanoparticles and nano-in-micro particles (NiMPs) with a Zetasizer Nano ZS (Malvern Instruments, Malvern, United Kingdom) utilizing a folded capillary cell DTS 1070 (Malvern Instruments, Malvern, United Kingdom). The instrument was calibrated routinely with a −50 mV latex standard. PLGA nanoparticles were suspended in Milli-Q water, as well as in 2-propanol as comparison. Due to initially occurring agglomeration and swelling of chitosan NiMPs in Milli-Q water, NiMPs were suspended and measured only in 2-propanol via ultrasonic treatment of 30 s in an ultrasonic bath. For measurements in aqueous media Smoluchowski approximation and Huckel approximation for non-aqueous measurements in 2-propanol was used. The measurements were repeated five times at neutral pH and 25°C after 600 s equilibration time.

### Encapsulation Efficiency

The encapsulation efficiency of nano-in-micro particles (NiMPs) was examined via image analysis from Confocal Scanning Electron Microscopy images using the software ImageJ (Wayne Rasband, 1997) and two different calculations. First, the average number of PLGA nanoparticles colored in red incorporated in green stained chitosan microparticles was determined using the assumption of particles equal in size with the mean diameters extracted from light scattering measurements for nanoparticles and microparticles, respectively (*n* = 5). In the second calculation, the relative encapsulation efficiency was observed relating the total cross-section area of PLGA nanoparticles (red) to the cross-section area of nanoparticles solely outside of chitosan microparticles (*n* = 5). Consequently, the difference between total cross-section area of nanoparticles and cross-section area of nanoparticles outside of microparticles revealed the percentage of successfully encapsulated nanoparticles through spray drying.

### Tissue Preparation

Pig snouts were purchased from a local slaughterhouse. Mucosa specimens were excised from the dorsal part of the *concha nasalis dorsalis* (Getty 1975; Mistry 2009). The olfactory mucosa was gently removed from the underlying cartilage with the help of forceps (Ladel et al., 2018). Excised specimens were further dissected into 1–2 cm^2^ samples. *Post mortem* delay of the mucosa was below 2 h.

### *Ex vivo* Permeation Studies

For particle application, prepared mucosa specimens were put into a Petri dish. ∼12 mg of NiMPs were administered to the olfactory mucosa, which due to the chitosan coating is equivalent to ∼4 mg of PLGA particles for NiMPs with low chitosan ratio and ∼2 mg for NiMPs with high chitosan content, examined from the actual encapsulation efficiency evaluated via image analysis from Confocal Laser Scanning Microscopy images (chapter 2.7). The mucosa was then placed into a modified side-by-side cell consisting of two micro reaction tubes (1.5 ml) between clamping tongs (Figure 3A), resulting with the inner radius 5 mm in a permeation area of 78.5 mm^2^. The bottom microtube was filled with 260 µl of phosphate-buffered saline (PBS) at pH 7.4 before placing the mucosa with its basolateral side on top of it. The system was sealed to avoid leakage by closing the cell. Finally, the cell was put upside down into an incubator at 35°C and 90% humidity to simulate the natural conditions within the nasal cavity for 5 min to 2 h. As a comparison, 20 µl of the PLGA nanoparticle suspension was applied similarly; however, due to the production procedure ∼0.2 mg particles were applied in this case, which does hence only allow a quantitative comparison among similar applied samples. Due to the sensitivity of PLGA nanoparticles they could not be dried and applied as a powder; however, the NiMPs could not be suspended in aqueous media due to the intense swelling of chitosan. Consequently, PLGA nanoparticles and NiMPs were treated differently in the experiments, which is considered in the comparison of the results. Control samples were taken respectively at time point 0 min directly after application. After the respective incubation times, the mucosa specimens were immediately fixed in 4 %wt. paraformaldehyde for at least 2 h and stored in 30%wt. sucrose at 4°C until sectioning. The tissue samples were cut in 20 µm slices at the site of applied particles in a cryostat at −25°C (HM560, Thermo Fisher Scientific, Dreieich, Germany) and mounted on Superfrost®Plus Micro slides (Thermo Fisher Scientific, Waltham, United States).

### Immunohistochemistry and Histological Staining

The structural integrity of the epithelial layer was confirmed by hematoxylin-eosin (HE) staining. HE-stained slides were dehydrated in ethanol and 2-propanol. Finally, they were embedded with Eukitt^®^ Quick-hardening mounting medium (Sigma-Aldrich, St. Louis, United States) and covered with a coverslip.

For observation via confocal laser scanning microscopy either solely cell nuclei were stained with DAPI (4′,6-Diamidin-2-phenylindol 2HCl; Serva Electrophoresis GmbH, Heidelberg, Germany) or DAPI staining was performed after the immunohistochemistry staining procedure. Tissue explants viability was verified with control samples live-stained after incubation up to 5 h with Hoechst 33342 (Cell Signaling Technology Inc., Danvers, United States). To visualize the initial immune reaction, CD14^+^ cells were stained via CD14 primary antibody (#NB100-77758, Novus Biologicals, Littleton, United States) and goat anti-mouse secondary IgG (H+L) Alexa Fluor^®^ 647 (#A-21235, Thermo Fisher Scientific, Waltham, United States). Neurofilament heavy protein (200 kDa) NF-H was marked to visualize co-localization with neuronal bundles and therefore stained with NF-H primary antibody (#PA1-10002, Thermo Fisher Scientific, Waltham, United States) and goat anti-chicken secondary IgY (H+L) FITC (#A16055, Thermo Fisher Scientific, Waltham, United States). Cell-cell junction protein 1 (*Zonula occludens* protein 1; *ZO-1*) was detected via *ZO-1* primary antibody (#NBP1-85047, Novus Biologicals, Littleton, United States) and secondary goat anti-rabbit IgG (H+L) Alexa Fluor^®^ 647 (#ab150083, Abcam, Cambridge, United Kingdom). The fluorescently stained samples were fixated with Fluoroshield™ mounting medium (Sigma-Aldrich, St. Louis, United States) and covered with a coverslip.

## Results

### Nanoparticles

#### Particle Size Distribution

The particle size of the poly(lactic-*co*-glycolic acid) PLGA nanoparticles manufactured with precipitation was adjusted by the variation of PLGA concentration in the solvent phase. 5 mg ml^−1^ PLGA starting concentration resulted in an average particle size of 81.6 nm with a Polydispersity index (PI) of 0.071, whereas characterization of the particles manufactured with 10 mg ml^−1^ PLGA starting concentration revealed a mean particle size of 174.4 nm and a PI value of 0.037. Hence, both samples produced via precipitation resulted in monodisperse size distributions below the critical value of 0.1 (Hughes et al., 2015). PLGA nanoparticles produced via the double emulsion method resulted in a medium size of 519.7 nm with a narrow size distribution characterized by a PI of 0.28.

#### Zeta Potential

The Zeta potential was characteristically negative for all prepared PLGA nanoparticles (Vila et al., 2002; Ravi Kumar et al., 2004). However, 2-propanol is less polar than water and has a lower dielectric constant of 18.0 As Vm^−1^ at 25°C compared to 78.5 As Vm^−1^ of water (Akerlof 1932), the resulting zeta potential in both investigated solvents did not differ significantly.

#### Morphology

**FIGURE 1:**
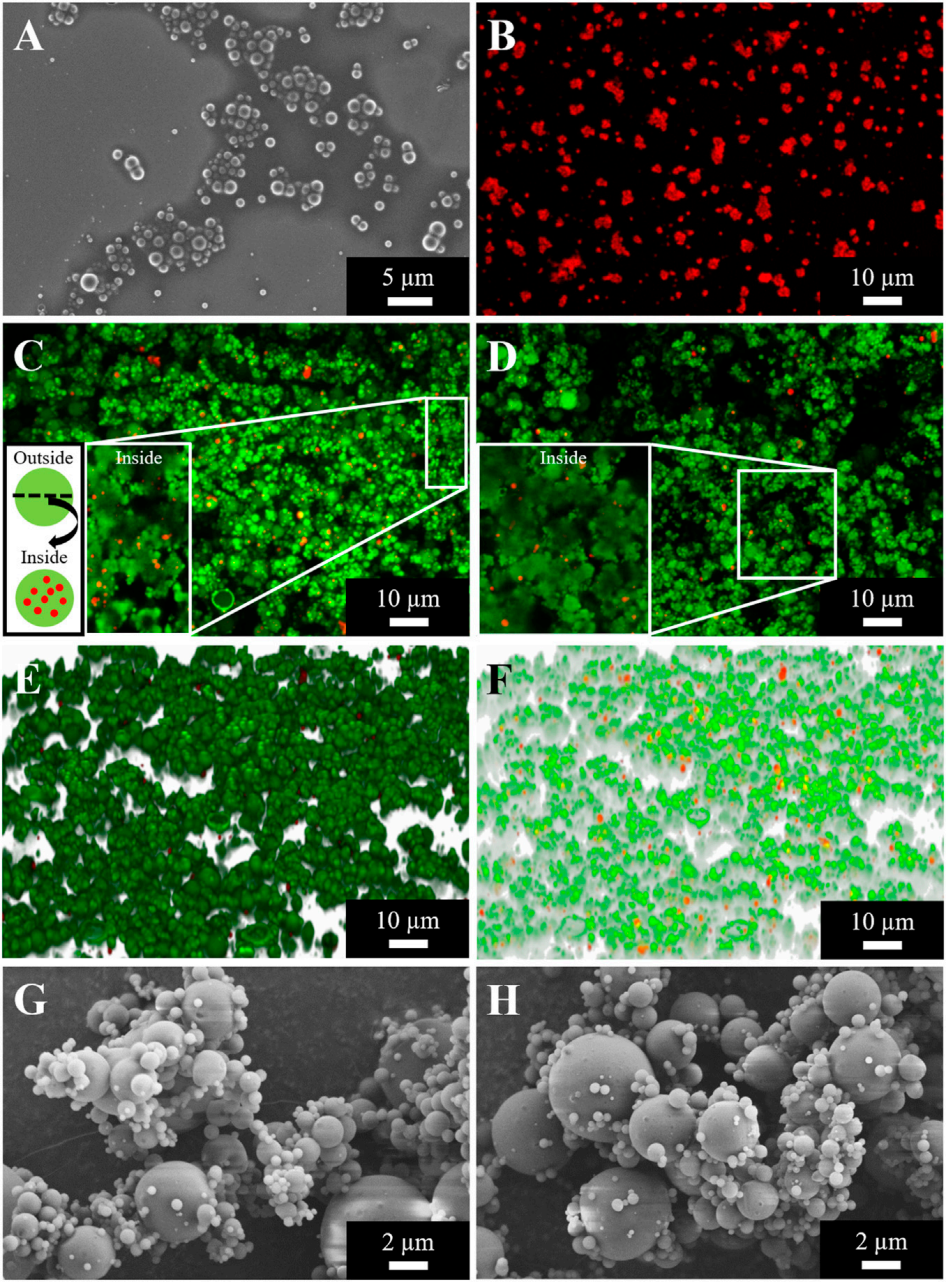
PLGA nanoparticles prepared by double emulsion technique **(A, B)** and spray dried nano-in-micro particles (NiMPs) prepared by spray drying at two different polymer ratios consisting of PLGA nanoparticles (520 nm) and chitosan as matrix material **(C–H)**. **(A)** Scanning Electron Microscopy (SEM) image, magnification 5 kX. **(B)** Confocal Laser Scanning Microscopy (CLSM) picture, mag. 630 X, maximum intensity projection (MIP) stiched together from a z-stack, particles labeled with Lumogen F Red. **(C)** CLSM image of NiMPs 1/3 chitosan:2/3 PLGA, mag. 630 X, MIP; Enlarged cross-section of particles and particle schematic; Chitosan (green) and PLGA nanoparticles (red). **(D)** CLSM image of NiMPs 2/3 chitosan:1/3 PLGA, mag. 630 X, MIP. **(E)** 3D projection of CLSM images (green: chitosan; red: PLGA) stiched together from a z-stack, mag. 630 X, external view. **(F)** Transparent 3D projection of CLSM images (green: chitosan; red: PLGA) stiched together from a z-stack, mag. 630 X, internal view. **(G)** SEM image of NiMPs 1/3 chitosan:2/3 PLGA, mag. 10 kX. H: SEM picture of NiMPs 2/3 chitosan:1/3PLGA, mag. 10 kX. 3D projections (external, transparent) of CLSM images from 2/3 chitosan:1/3 PLGA NiMPs displayed in Supplementary Figure S1.

PLGA nanoparticle morphology was examined using Scanning Electron Microscopy (SEM) and Confocal Laser Scanning Microscopy (CLSM). As presented in Figure 1A, nanoparticles prepared by the double emulsion technique are spherical and show a smooth surface. Further, their successful labeling with Lumogen F Red was confirmed with their intense and homogenous color (Figure 1B).

### Nano-in-Micro Particles

#### Particle Size Distribution

**FIGURE 2:**
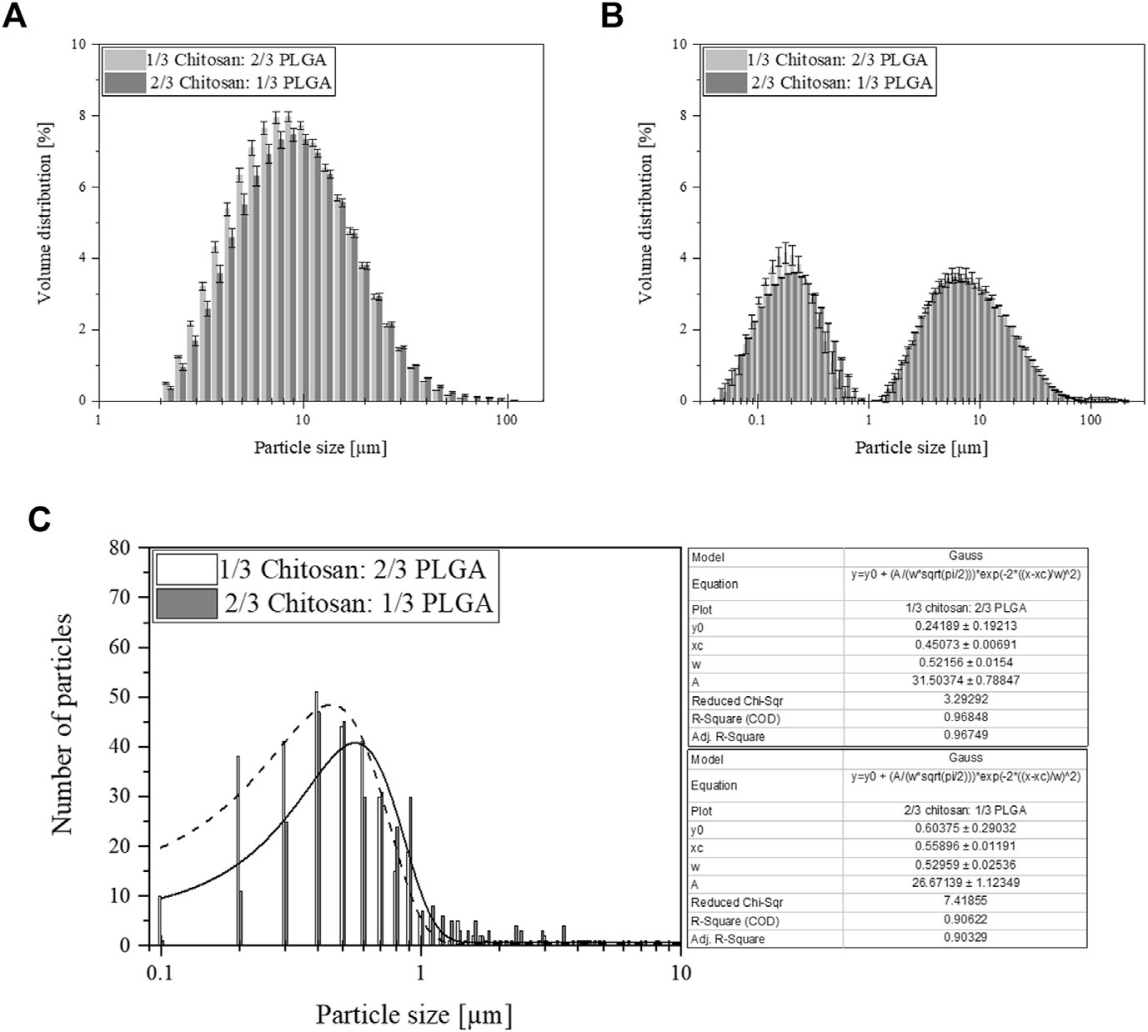
**(A)** Volume size distribution of nano-in-micro particles (NiMPs) produced via spray drying. Particles consist of PLGA nanoparticles (520 nm) coated with chitosan at a polymer ratio of either 1/3:2/3 or 2/3:1/3; (*n* = 5). **(B)** Volume size distribution of NiMPs after 1 min ultrasonic treatment in the measurement cell; (*n* = 5). **(C)** Particle number distribution (*n* = 307 particles) extracted from Scanning Electron Microscopy (SEM) images and fitted with a Gaussian fit; Dashed line (1/3 chitosan:2/3 PLGA) and full line (2/3 chitosan:1/3 PLGA).

Herein prepared nano-in-micro particles (NiMPs) consisting of PLGA nanoparticles (520 nm) coated via spray-drying with chitosan varying the chitosan:PLGA polymer ratio. First, NiMPs with 1/3 chitosan:2/3 PLGA were prepared and analyzed via static light scattering (SLS), while stirring at 1,500 rpm in the µP2000 measurement cell. The resulting Gaussian shaped volume size distribution is presented in Figure 2A. Compared to the NiMPs with 2/3 chitosan:1/3 PLGA, the particle size increased with a higher chitosan ratio and the histogram is; therefore, shifted to the right towards bigger particle sizes.

Further, we calculated the d(0.5) value for both types of NiMPs representing 50% of the particle populations respectively, as well as the mean particle size and thereof the Polydispersity Index (PdI) to characterize the homogeneity of the particle population. These values are presented in Table 1. NiMPs with a higher chitosan ratio resulted in a d(0.5) particle size of 8.65 µm (Table 1) compared to the slightly smaller particles with less chitosan characterized by a d(0.5) diameter of 8.10 µm. Both spray-dried samples resulted in a unidisperse particle size distribution; however, the population of NiMPs with less chitosan is more homogenous than the sample with a higher chitosan ratio, characterized by a smaller PdI (Table 1).

**TABLE 1:** Characteristic parameters of nano-in-micro particles (NiMPs) obtained from static light scattering measurements. d(0.5) value represents the diameter of 50% of the particle population. The Polydispersity index (PdI) characterizes the homogeneity of the particle population; 1 min ultrasonic treatment (US).

Method	SLS	SLS	SLS	SEM
Condition	Agglomerated suspension in 2-propanol	Suspension in 2-propanol	Suspension in 2-propanol	Dry
Treatment	1,500 rpm	After US (First Peak)	After US (Second Peak)	None
d [µm] 1/3 chitosan: 2/3 PLGA	8.10	0.18	6.61	0.45
d [µm] 2/3 chitosan: 1/3 PLGA	8.65	0.21	5.75	0.56
PdI 1/3 chitosan: 2/3 PLGA	0.99	0.70	1.89	0.6
PdI 2/3 chitosan: 1/3 PLGA	1.17	0.87	1.84	1.3
Distribution	Monodisperse	Bidisperse	Bidisperse	Monodisperse

Second, the samples were treated with ultrasound for 1 min in the µP2000 measurement cell and afterwards measured again at 1,500 rpm. The second measurement cycle revealed a bidisperse size distribution for both sample types displayed in Figure 2B. The volume fraction is divided equally in a nanoparticle and a microparticle fraction, due to the split up of agglomerates. The nanoparticle fraction of NiMPs with low chitosan ratio (1/3 chitosan:2/3 PLGA) is characterized by a mean particle diameter of 0.18 µm compared to 0.21 µm of NiMPs with high chitosan ratio (2/3 chitosan:1/3 PLGA) (Table 1). Due to the previously described mean size of 519.7 nm of PLGA nanoparticles, the nanoparticle fractions of both NiMP samples should mainly consist of pure chitosan. Further, the nanoparticle fraction resulted in slightly bigger particles due to the higher chitosan ratio and the size distribution of this fraction is broader (Table 1). In contrast, the microparticle fraction of NiMPs with less chitosan is characterized by a mean particle diameter of 6.61 µm, whereas NiMPs consisting of more chitosan have a smaller mean diameter of 5.75 µm. Additionally, the microparticle fraction of NiMPs with high chitosan ratio is narrower than the microparticle fraction of NiMPs with low chitosan ratio (Table 1). These findings imply that additional chitosan in the spray drying process is mainly forming pure chitosan nanoparticles, not NiMPs. Therefore, it is important to compare the encapsulation efficiencies and understand, whether a higher matrix ratio is beneficial to encapsulate nanoparticles via spray drying, which is explained below.

Additionally, we measured the size distribution of NiMPs in representative Scanning Electron Microscopy (SEM) images using ImageJ software and plotted their resulting diameters against frequency. The following size distributions are presented in Figure 2C together with a non-linear Gaussian fit performed in OriginPro software. The majority of 1/3 chitosan:2/3 PLGA-NiMPs has a diameter of 0.45 µm. NiMPs with high chitosan ratio (2/3 chitosan:1/3 PLGA) resulted in a slightly greater mean diameter of 0.52 µm (Table 1), which confirms that more chitosan available during spray-drying as matrix material results mainly in bigger nanoparticles. Further, the displayed size distribution is monodisperse; however, very broad. This additional analysis shows, NiMPs (2/3 chitosan:1/3 PLGA) consisting of more chitosan result in a broader size distribution than NiMPs produced with less chitosan (1/3 chitosan:2/3 PLGA), which is attributed to the smaller sample size of the image analysis. In the size distribution extracted from SEM images further only few microparticles occur. In contrast to this, the size distributions examined via static light scattering represent the samples volume fraction. In volumetric size distributions, few bigger particles represent a bigger fraction than in frequency displaying distributions, where the volume of the measured particles within the sample is not considered.

#### Zeta Potential

The examination of chitosan NiMPs in contrast to PLGA nanoparticles revealed an overall positive zeta potential, which is characteristically for chitosan (Vila et al., 2002; Ravi Kumar et al., 2004) and proves the successful encapsulation of PLGA nanoparticles into the chitosan matrix by spray drying.

#### Encapsulation Efficiency

Comparing both spray-dried NiMP samples with different polymer ratios, in NiMPs with less chitosan and higher PLGA ratio (1/3 chitosan:2/3 PLGA) generally about seven 519.7 nm PLGA nanoparticles (NP) per single chitosan 6.61 µm microparticle (MP) have been encapsulated (Figure 1C). Red nanoparticles are distributed inside of green chitosan microparticles (Figure 1F). Solely, a few nanoparticles remained on the surface (Figure 1E) and hence most nanoparticles were encapsulated successfully.

Due to the higher chitosan ratio NiMPs with 2/3 chitosan:1/3 PLGA showed an average of only two 519.7 nm PLGA NP in one chitosan 5.75 μm MP (Figure 1D). Both samples further resulted in similar encapsulation efficiencies; however, NiMPs with a lower chitosan ratio were produced with proportionally less matrix material to cover PLGA nanoparticles. NiMPs consisting of 1/3 chitosan:2/3 PLGA resulted in an encapsulation efficiency of 39 ± 19% (Figure 1C). NiMPs with a higher chitosan ratio (2/3 chitosan:1/3 PLGA) yielded in a higher encapsulation efficiency of 51 ± 16% (Figure 1D), however this is not significant. Consequently, PLGA nanoparticles were successfully encapsulated into chitosan via spray drying, but a 1/3 higher matrix composition did not significantly improve the encapsulation efficiency. Proportionally, even more nanoparticles were encapsulated with a lower chitosan amount.

#### Morphology

NiMPs were further investigated via Scanning Electron Microscopy (SEM). Exemplary pictures, at a magnification of 10 kX, are displayed in Figure 1E (1/3 chitosan:2/3 PLGA) and Figure 1F (2/3 chitosan: 1/3 PLGA). The previously-described effect, causing slightly bigger particles due to the increase in chitosan ratio during spray drying, can also be supported by the SEM analysis. Samples prepared with a ratio of 1/3 chitosan:2/3 PLGA show more particles below one micron (Figure 1G), which is additionally proved with the size distribution in Figure 2C. In contrast, spray-dried NiMPs with the opposite polymer ratio (2/3 chitosan:1/3 PLGA) are slightly bigger (Figure 1H, Figure 2C).

Further, NiMPs are, independent of their polymer ratio, spherically shaped and show a smooth surface, which can be attributed to the spray drying inlet temperature of 100°C and a resulting outlet temperature of 45 ± 1°C, which allows continuous evaporation of the water phase and results in homogenous particles with dense pores and hence smooth appearing surface (Paudel et al., 2013).

### Mucosal Uptake of PLGA Nanoparticles

**FIGURE 3:**
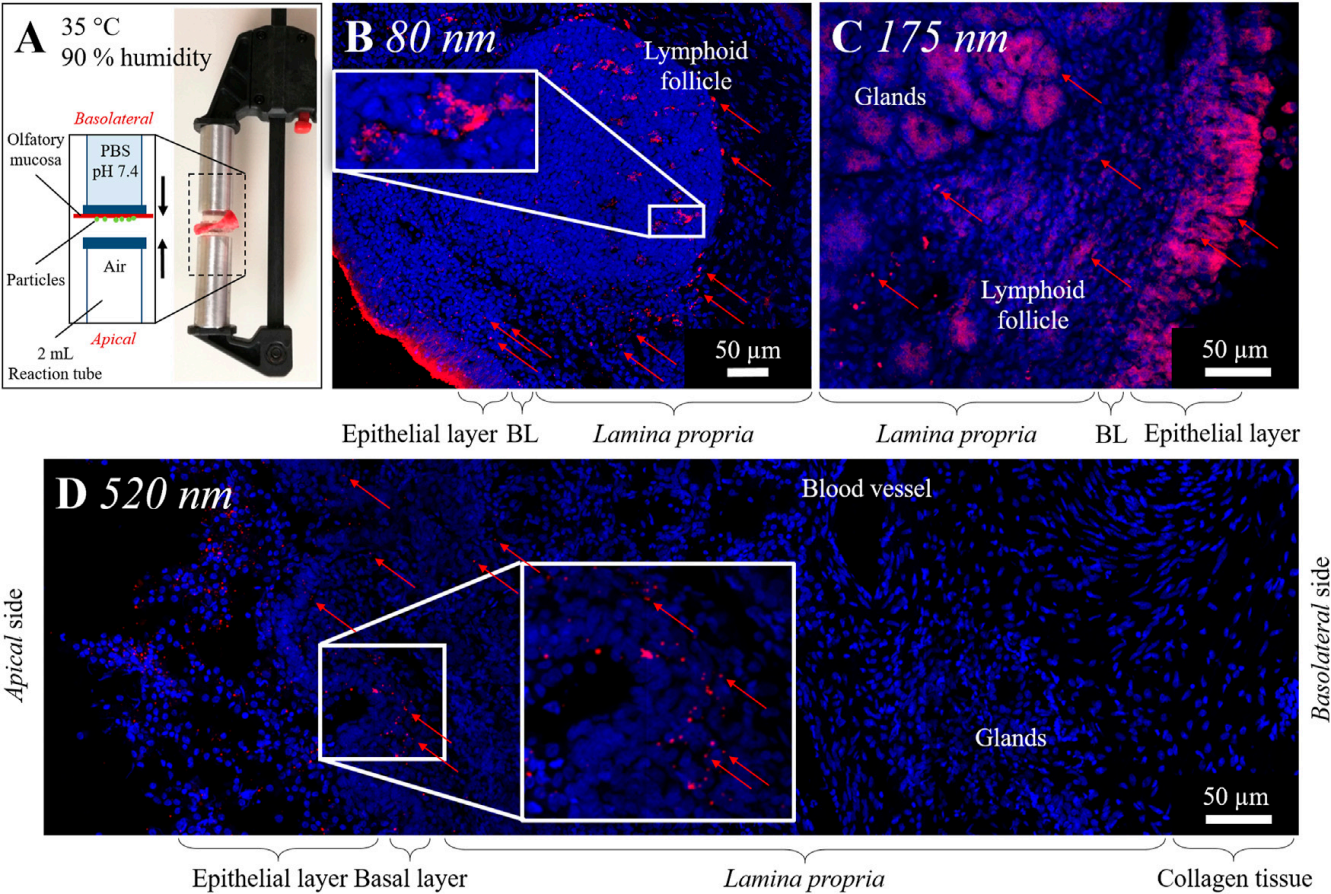
**(A)** Experimental setting of *ex vivo* particle permeation experiments, porcine olfactory mucosa incubated in specialized side-by-side cell at physiological conditions of the nasal cavity (35°C, 90% humidity). **(B–D)** Uptake of PLGA nanoparticles (red) 5 min **(B, C)** and 10 min **(D)** after application to the apical side of the olfactory mucosa. Maximum intensity projection (MIP) of Confocal Laser Scanning Microscopy (CLSM) images stitched together from a z-stack. Cell nuclei stained with DAPI (blue). PLGA nanoparticles colored with 14 μg ml^−1^ (80 nm), 28 μg ml^−1^ (175 nm), or 28 μg ml^−1^ (520 nm) Lumogen. Red arrows mark single particles. Area of particle accumulation enlarged.

In this study, we examined the uptake of PLGA nanoparticles with diameters of 80, 175, and 520 nm into olfactory mucosa tissue. Therefore, nanoparticle suspensions have been applied onto the apical site of explanted porcine mucosa specimens and incubated in specialized side-by-side cells at physiological conditions of 35°C and 90% humidity (Figure 3A) mimicking the nasal cavity. Quality control of olfactory mucosa biopsy and further processing steps including cutting, particle application, fixation, and cryo-sectioning occurred via hematoxylin-eosin (HE) staining. Only specimens with an intact epithelial layer were analyzed; those with a damaged epithelial layer were excluded from the study because a compromised epithelial layer could enhance particulate uptake. Further, sample thickness of *ex vivo* specimens was 1,273 ± 356 μm, whereas individual samples were not significantly different in thickness (Shaipiro-Wilk normality test, One-Way-ANOVA with Levene’s variance test and Bonferoni posthoc test; *α* = 0.05 respectively). Detailed thickness data and statistical analysis are presented in Supplementary Figure S2.

PLGA nanoparticles, with a diameter of 80 and 175 nm have been taken up immediately into the *lamina propria* of the olfactory mucosa within 5 min after application. Particles are spread over the *lamina propria*. 80 nm nanoparticles, co-localized with cell nuclei, have been observed in lymphoid follicles (Figure 3B) and the epithelial cell layer, which implies spontaneous cellular uptake of these nanoparticles. 175 nm particles have additionally been documented more prominent in glands than in lymphoid follicles (Figure 3C).

In general, it is reported that cells can take up nanoparticles, with a diameter below 200 nm, intracellularly after intranasal administration (Mistry et al., 2009; Bourganis et al., 2018; Rabiee et al., 2020). Muntimadugu et al. (2016) has further already reported the transport of PLGA nanoparticles, with diameters below 200 nm, along axons within the nasal mucosa (Muntimadugu et al., 2016). This was also proven within our experiments for porcine olfactory mucosa. We further investigated PLGA nanoparticles with a 520 nm diameter. In contrast to intranasal application, K. Y. Win and S. S. Feng et al. (2005) proved the intracellular uptake of 500 nm polymer particles into Caco-2 epithelial cells (Win and Feng 2005). In our study, 10 min after application to the olfactory mucosa explants, cells also seemed able to take up 520 nm nanoparticles; these were found distributed in the epithelial layer and mainly in the outer 150 µm of the *lamina propria* (Figure 3D). Overall, the uptake of the larger-size particles occurred in slower motion compared to 80 and 175 nm particles. Further, 520 nm particles have not, in contrast to smaller nanoparticles, been observed primarily near nuclei within the first 15 min but between, hence intercellularly. Within 15 min, further 520 nm particles have been detected only sparely in glands, not in deeper layers of the *lamina propria*, nor in the collagen tissue, which connects the porcine mucosa with the nasal septum *in vivo*.

An overview of size- and time-dependent PLGA nanoparticle uptake and distribution is presented in Table 2:

**TABLE 2:** Size- and time-dependent uptake, as well as co-localization with sub structures in the olfactory mucosa, of PLGA nanoparticles (suspension) and spray dried nano-in-micro particles (NiMPs) (powder) consisting of 520 nm PLGA particles embedded in chitosan. 5 min, 10 min, or 15 min after particle application to the apical side and incubation at physiological conditions of the nasal cavity (35°C, 90% humidity). (+) rarely, (++) intermediately, (+++) often particles observed, or (−) no particles observed. Examined samples: PLGA nanoparticles (*n* = 3), respectively; NiMPs (*n* = 6). Quantitative comparison only applicable for PLGA nanoparticles among each other and NiMPs separately due to different applied particle amounts (suspension versus powder).

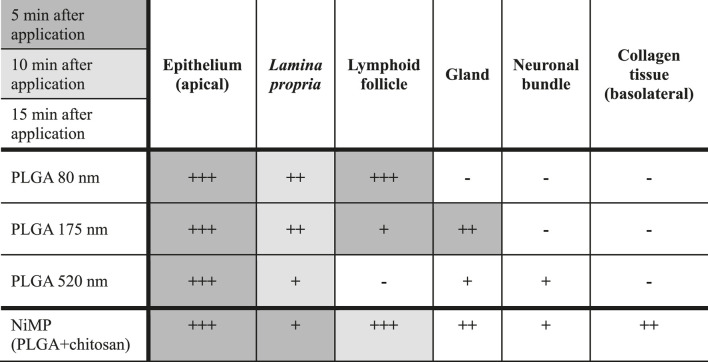

In summary, the uptake of PLGA nanoparticles, displayed in Table 2, is fast, within only 5 min after application to the apical side of olfactory mucosa, of PLGA nanoparticles up to a diameter of 520 nm, into the epithelial cell layer. Further size dependent penetration occurs within 10 min to the underlying *lamina propria*. Herein, 80 nm particles penetrate to a high extent, whereas similar amounts of 175 nm particles were taken up. 520 nm, in contrast, penetrated to a very low extent and, hence, were also slower. Further, many 80 nm particles were detected within 5 min, especially intracellularly within lymphoid follicles, which can imply an interaction with immune cells. In contrast, 175 nm particles were only rarely found associated with lymphoid follicles and 520 nm particles were, even within 15 min, not detected co-localized with lymphoid follicles. This indicates a size dependent relation in transport. The initial immune reaction was investigated within this study and is described below. Supplementary, 175 nm particles have been detected after 5 min in glands, 520 nm particles only after 15 min, and 80 nm particles were not detected at all. This again implies a size dependent differentiation in transport routes, but does not impact transport towards the brain or the central nervous system (CNS). In contrast, gland associated nanoparticles might stuck in the mucosa and are, therefore, less effective as drug delivery system. Additionally, only 520 nm nanoparticles have been detected in neuronal bundles, which could enable further transport to the brain or the CNS. Consequently, especially 520 nm particles seem to have the ability for transcellular neuronal transport, in contrast, 80 and 175 nm do not. Those seem to be transported only transcellular across other cell types. Finally, none of the investigated pure PLGA nanoparticle samples were detected within the basolateral collagen tissue and, thereof, none of these penetrated the whole transverse section of the tissue samples within 15 min following application.

### Nano-in-Micro Particle Uptake

PLGA nanoparticles, with a 520 nm diameter, were embedded in a second production step via spray drying in chitosan. The previously-described variation of polymer ratio PLGA:chitosan showed no impact on olfactory uptake. Therefore, the following results are true for all, herein produced and described, nano-in-micro particles (NiMPs). ∼12 mg NiMPs were applied onto porcine olfactory mucosa specimens and incubated similarly to pure nanoparticles, in a side-by-side cell at 35°C and 90% humidity, mimicking the nasal cavity (Figure 3A). Due to the different application of PLGA nanoparticle suspensions as powder here, only the occurrence of NiMPs and chitosan-coated PLGA particles is examined, but not the quantitative particle amount. This is because more PLGA particles have been applied, respectively, on powder samples.

**FIGURE 4:**
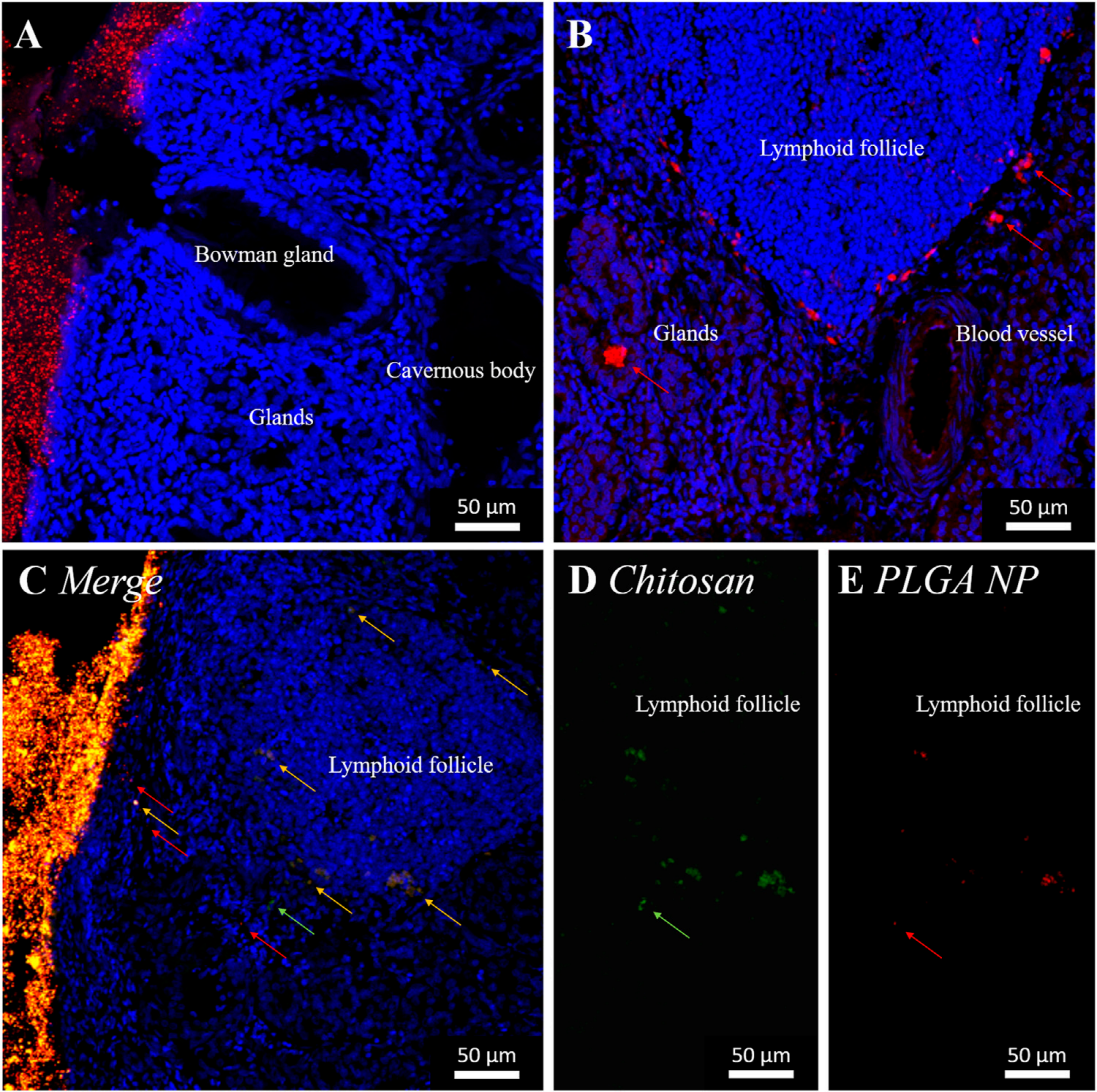
Permeation of nano-in-micro particles (NiMPs) consisting of PLGA nanoparticles (520 nm; red) encapsulated in chitosan applied to the apical side of porcine olfactory mucosa. Maximum intensity projection (MIP) of Confocal Laser Scanning Electron Microscopy (CLSM) images stitched together from a z-stack. Cell nuclei stained with DAPI (blue). Images taken after 30 min **(A)** or 2 h **(B–E)**. **(C)** Merged image shows overlapping signals of nanoparticles (red) and chitosan labeled with fluorescein (green) appearing yellow. **(D)** Corresponding single channel of chitosan (green). **(E)** Corresponding single channel of nanoparticles (red). (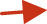) PLGA nanoparticles, (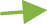) chitosan, (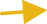) co-localized PLGA nanoparticles and chitosan. PL, Particle layer; BL, Basal layer.

The applied particle patch (red) covered the epithelial layer completely (Figure 4). In contrast to the above, chapter 3.3 described the distribution of purely applied 520 nm PLGA particles in the outer ∼150 µm of the *lamina propria*, chitosan-coated NiMPs have been detected 15 min after application spread deep within the *lamina propria* even distributed over the entire width of the olfactory mucosa sample reaching the collagen tissue at the basolateral side (Supplementary Figure S3). All findings of NiMP-uptake within the initial 15 min after application to the olfactory mucosa are summarized and displayed in Table 2.

Due to the complete coverage of the epithelial layer with the applied particle layer, initially within 5 min, many NiMPs were found associated with the epithelium. Fewer NiMPs were found in the *lamina propria*; however, the penetration occurred faster than for pure 520 nm PLGA particles without chitosan coating. Within 10 min, NiMPs were mainly detected associated with lymphoid follicles (Supplementary Figure S4). After 15 min, NiMPs were finally detected in glands and neuronal bundles to a slightly lower extent. Finally, within 15 min, NiMPs were detected spread over the whole transverse section of the mucosal tissue sample, even distributed at the basolateral side in the collagen tissue. Consequently, the chitosan-coating herein accelerated the uptake of NiMPs, compared to the identical purely-applied PLGA particles, with 520 nm in the initial 15 min after application. However, to analyze the quantitative amount further investigations are needed, due to the different particle amounts applied, caused by the sampling procedure.

30 min following particle application, surface located bowman glands have been detected, where the gland-overlaying particle patch was cleared (Figure 4A and Supplementary Figure S6). Therefore, epithelial glands seem to be able to either take up particles within 30 min, resulting in enhanced uptake compared to the surrounding epithelial layer, or transport overlying particles away due to the ongoing mucus production mechanism (Sadé et al., 1970; Aspden et al., 1995; Aspden et al., 1997). For this reason, we additionally stained control samples, after 5 h incubation, with Hoechst 33342 dye, which solely stains intact cell nuclei to prove cell viability within and after the experiments. This live-stain experiment proved that cells of the excised mucosa samples were alive throughout the whole duration of the experiments (data not shown). As a result, it could be assumed that the monitored glands were also able to produce mucus during the experiment and, therefore, possibly transported overlying particles away.

Samples examined 2 h after particle application showed similar results to the above described results 15 min following application. NiMPs were observed over the whole transverse section of the porcine olfactory mucosa, including collagen tissue layers at the basolateral side of the sample (Supplementary Figure S5). Furthermore, 520 nm particles were detected in mucus-producing glands within the tissue (Figure 4B), and in the outer zone of lymphoid follicles (Figures 4B–E). Therefore, NiMPs also seem to be transported to lymphoid follicles, as similar behavior was found after 5 min for nanoparticles with 80 nm diameter. Due to the green labeling of chitosan in NiMPs, we were able to additionally prove the co-localization of chitosan (Figures 4C,D) with PLGA nanoparticles (red) (Figures 4C,E) in the outer zone of lymphoid follicles. Consequently, not only the pure PLGA particles were transported there but also chitosan-coated NiMPs. Co-localization with glands (Figure 4B) could also be attributed to the mucoadhesive properties of chitosan (Henriksen et al., 1996). When passing glands, mucus in these glands seems to hold up chitosan-coated nanoparticles due to mucoadhesion.

### Influence of Chitosan Coating

In general, the interaction of chitosan and PLGA nanoparticles within mucosal tissues is not yet well characterized. We, therefore, examined the following immunohistochemistry studies and compared purely applied 80, 175, and 520 nm PLGA particles with nano-in-micro particles (NiMPs) consisting of 520 nm PLGA nanoparticles embedded in chitosan via spray drying.

#### Co-Localization With Initial Immune Cells and Neuronal Fibers

**FIGURE 5:**
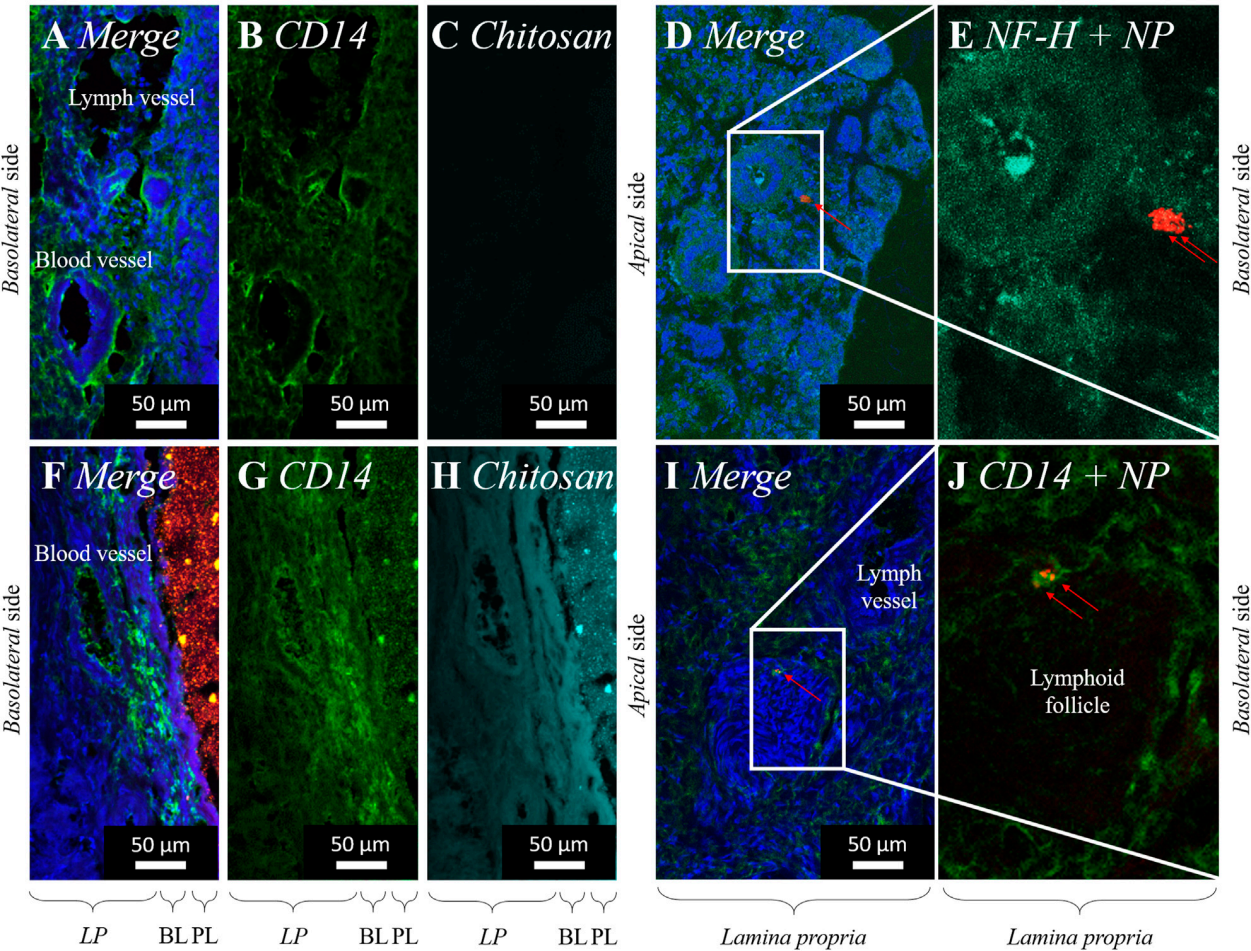
Permeation of PLGA nanoparticles (520 nm; red) **(A–E)** and PLGA-chitosan nano-in-micro particles (NiMPs) **(F–J)**. Maximum intensity projection (MIP) of Confocal Laser Scanning Microscopy (CLSM) images stitched together from a z-stack, taken 15 min after application to the apical side of porcine olfactory mucosa. Cell nuclei stained with DAPI (blue). Red arrows mark nanoparticles (red) within the tissue. CD14 (green) labelled with anti-mouse Alexa Fluor^®^ 647 secondary antibody **(A, B, F, G)**. NF-H (cyan) labelled with anti-chicken FITC secondary antibody **(D, E)**. Chitosan labeled with fluorescein (cyan) **(H)**. Overlapping signals of red and green appear yellow **(F)** due to co-localization of PLGA nanoparticles (red) and chitosan (green). PLGA nanoparticles (single red channel) displayed in Supplementary Figure S6B, C. LP, Lamina propria; BL, Basal cell layer; PL, Particle layer; EL, Epithelial cell layer.

When pure PLGA nanoparticles were applied to the porcine olfactory mucosa sample, several nanoparticles were taken up into the epithelial layer, these also passed the basal cell layer (BL), and permeated into outer layers of the *lamina propria* (*LP*) as already described in the previous chapter. We additionally labeled CD14^+^ cells via immunofluorescence staining. As shown in Figure 5A (merge) and Figure 5B (single channel), CD14 was mostly detected homogenously distributed within the olfactory mucosa, since this biological barrier is an important immunologically-active tissue. Further, an increased signal was detected, particularly within blood vessels and lymphatic vessels, because CD14^+^ monocytes roll along with endothelial cells and are moving from the immunogenic active tissue site into the bloodstream. Due to the application of pure 80, 175 and 520 nm PLGA nanoparticles without chitosan, no chitosan signal was detectable in this kind of sample, which serves as a control here (Figure 5C).

Significant agglomerates of several nanoparticles have been further observed near nuclei (Figure 5D), 520 nm particles especially in neuronal bundles (Figure 5E) stained against the marker neuronal filament heavy protein (NF-H). Neuronal bundles are axonal fibers of primary neurons that merge in the *lamina propria* and connect the olfactory mucosa via the olfactory or the trigeminal nerve directly to the brain (Morrison and Costanzo 1990; Morrison and Costanzo 1992).

When chitosan-coated NiMPs have been applied to the porcine olfactory mucosa, within 15 min several chitosan-coated nanoparticles were also detected in the outer area of the sample, including the epithelial layer, basal cell layer (BL), and the outer layers of the *lamina propria* (*LP*) (previous chapter). NiMPs have also been observed in neuronal bundles (data not shown) and could, therefore, possibly be also transported along the olfactory and trigeminal nerve to the brain and the central nervous system (CNS). As stated in chapter 3.4, we have already proved that not only pure PLGA nanoparticles can penetrate the olfactory mucosa, but also chitosan associated with these nanoparticles, and even to a faster extent. In Figure 5H, we solely displayed the penetration of chitosan labeled with fluorescein, which indicates, that chitosan is also able to dissociate from PLGA nanoparticles and independently penetrate the outer ∼150 µm of the olfactory mucosa within 15 min after application to the apical side of the sample. Further, an intensity gradient of chitosan is visible decreasing to deeper layers within the tissue.

In the examination of CD14^+^ cells after application of chitosan-coated NiMPs, a CD14^+^ signal was detected on the surface of single epithelial cells; however, the epithelial layer herein was completely covered by the particle patch (Figure 5F). Further, CD14^+^ monocytes were again detected at the inner wall of blood vessels. Additionally, an accumulation of CD14^+^ immunoreactivity was monitored in the outer layers of the *lamina propria* (Figure 5G), where no blood or lymphatic vessel is present. Moreover, in Figure 5I, we found a CD14^+^ signal surrounding a lymphoid follicle present in the *lamina propria*; especially one CD14^+^ cell directly associated with NiMPs on its surface, as well as intracellularly, seems to express CD14 on its cell surface (Figure 5J). In contrast to this, direct cellular CD14-asscociation with pure 520 nm PLGA particles could not be found. Bringing this together with the described chitosan penetration gradient (Figure 5H), we conclude that an attraction of immune cells occurred due to the presence of chitosan recognized by CD14^+^ monocytes or macrophages. This could be assigned to the lipopolysaccharide-receptor, which CD14 is part of, resulting in either a pro-inflammatory event triggered by M1-macrophages, or a wound healing procedure described by the increased presence of M2-macrophages (Davis et al., 2018).

#### Cell Junction Opening

Chitosan can open tight junctions (Fazil et al., 2012; Sonaje et al., 2012; Rassu et al., 2016), which are the characteristic intercellular connections present in epithelial cells and endothelial cells. We, therefore, stained the prominent component *Zonula occludens* protein 1 *ZO-1* via immunohistochemistry.

**FIGURE 6:**
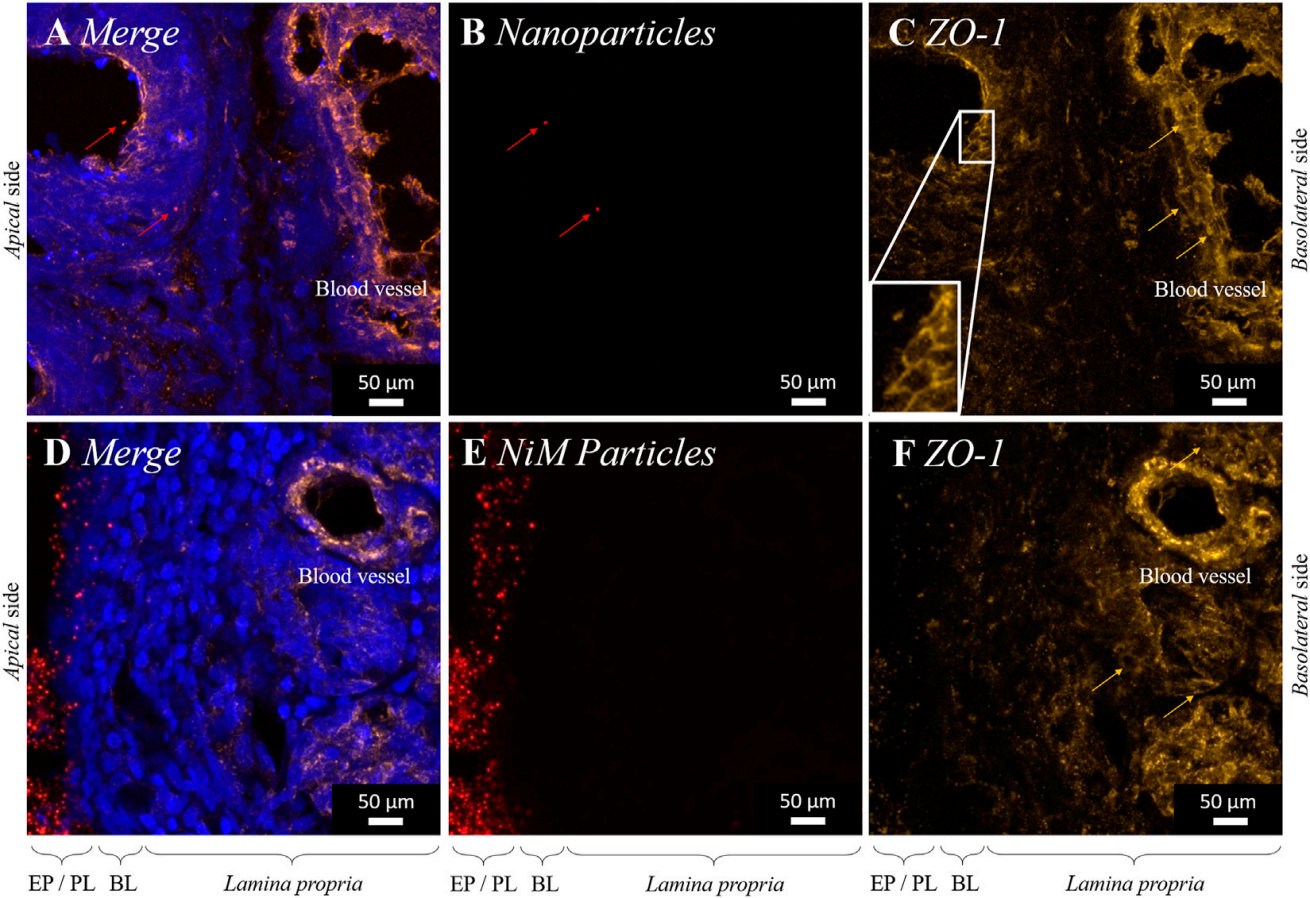
Permeation of PLGA nanoparticles (520 nm; red) **(A, B, C)** and PLGA-chitosan nano-in-micro particles (NiMPs) **(D, E, F)**. Maximum intensity projection (MIP) of Confocal Laser Scanning Microscopy (CLSM) images stitched together from a z-stack, taken 15 min after application to the apical side of porcine olfactory mucosa. Cell nuclei stained with DAPI (blue). *Zonula occludens* protein 1 (*ZO-1)* (amber) labeled with anti-rabbit Alexa Fluor^®^ 647 secondary antibody **(A,C,D,F)**. EL, Epithelial cell layer; PL, Particle layer; BL, Basal cell layer.

As already described in chapter 3.3, within 15 min after application PLGA nanoparticles (520 nm) permeated into the olfactory tissue sample (Figures 6A,B). *ZO-1* protein was detected mainly between epithelial cells forming the well-known tight junctions (Figures 6A,C). When pure PLGA nanoparticles have been applied, higher magnification revealed characteristically honeycomb-like staining resulting from intercellular present *ZO-1*, which was earlier described in cell culture (Stevenson et al., 1986; Anderson et al., 1988). The underlying *lamina propria* consists, to large extent, of fibroblastic cells, where *ZO-1* is mainly localized in endothelial cells. The localization in blood vessels was also proven in this study (Figures 6A,D). Open tight junctions are characterized by another conformation than in the closed stage. The membrane protein *ZO-1* is internalized into the cytoplasm, resulting in loosening of the intercellular connection (Smith et al., 2004). Consequently, due to the internalization of the former membrane protein, immunohistochemically targeting of *ZO-1* in the open conformation is hence not possible anymore, which results in discoloring.

Comparing the results of nanoparticle permeation to the application of chitosan-embedded NiMPs, we not only detected the above described uptake of PLGA nanoparticles associated with chitosan polymer molecules, we additionally displayed the ability of chitosan to penetrate the *lamina propria* dissociated from nanoparticles, approximately 150 μm, within 15 min after particle application to the olfactory epithelium (Figure 5H). Additionally, we investigated the presence of *ZO-1* signal in tight junctions. Following the application of NiMPs, we again detected a gradient signal (Figure 6F) reverse to the above-shown chitosan gradient (Figure 5H); therefore, matching the previously-described chitosan penetration of approximately 150 µm into the *lamina propria*, which caused *ZO-1* internalization and tight junction opening. Foremost in deeper layers of the *lamina propria, ZO-1* signal was still apparent proving working cell-cell junctions. Especially in the wall of blood vessels, the *ZO-1* immunoreactivity (Figures 6D,F) indicated still active tight junctions. Epithelial cells, which directly underlie the applied NiMPs faded also within 15 min and were therefore found discolored (Figures 6D,F) caused by chitosan mediated tight junction opening.

## Discussion

In this study, we successfully prepared poly(lactic-*co*-glycolic acid) PLGA nanoparticles of different sizes (80, 175, and 520 nm) using the precipitation and double emulsion method. Further, we produced specialized nano-in-micro particles (NiMPs) via spray drying, consisting of PLGA nanoparticles (520 nm), embedded into the matrix polymer chitosan.

NiMPs have in this study been produced via spray drying. In contrast to other innovative methods for nano- and microparticle production, e.g. electrospraying or supercritical CO_2_ assisted electrohydrodynamic processes (Jaworek and Sobczyk 2008; Baldino et al., 2019a; Baldino et al., 2019b), spray drying is an even commercially used formulation process. Especially the pharmaceutical industry (Stahl 1980; Lee et al., 2002) benefits from high throughput production of spray drying, concurrently resource-friendly, as well as from high resulting encapsulation efficiencies of up to 100% (Walz et al., 2018). Spray drying of chitosan has been already reported in the literature; however, ineffective process parameters or inhomogeneous particles have mainly been documented (He et al., 1999; Tokárová et al., 2013). To achieve a robust production process and reproducible particles, with homogenous size distribution and morphology, it is necessary to choose suitable process parameters, such as inlet temperature, feed rate, and gas flow (Paudel et al., 2013). The crucial factors in this process are droplet atomization followed by feed evaporation. To generate homogenous particles, atomization should be uniform, resulting in fine droplets at the first stage. These droplets then need to evaporate continuously in the second step, to achieve homogenous particles with smooth surface. Droplets dry from the outer to the inner phase, meaning that the remaining liquid in the center of the particle needs to evaporate through the already partly dried matrix. When evaporation takes place too fast at inlet temperatures above 100°C, either hollow particles are formed through the so-called Rush-hour effect (Paudel et al., 2013), or they even burst and empty shells remain. Contrary to common knowledge, wrinkled surfaces of spray-dried chitosan particles do not occur from the polymeric properties of chitosan itself, but mainly from the process parameters of spray-drying. When particles cool down at the end of the process, they shrink and characteristic wrinkles occur. Hence, it is possible to prepare reproducible smooth chitosan particles utilizing suitable spray drying parameters like the ones reported herein. With moderate evaporation rates, dense and homogenous particles were achieved. Consequently, we developed a robust spray drying process. The morphology of all particulate samples in this study was spherical, with smooth surfaces because we optimized the spray drying process performed with a Büchi B-290 Mini-Spray dryer. An inlet temperature of 100°C was identified, as a crucial parameter, which resulted in an outlet temperature of 45 ± 1°C. This allowed the polymer droplets to continuously evaporate, ultimately resulting in homogenous and smooth particles.

For the chitosan:PLGA polymer ratio of NiMPs, a higher chitosan ratio resulted in increased particle diameters determined via image analysis of Scanning Electron Microscopy images and static light scattering; however, NiMPs tend to agglomerate in suspension. They were split up during static light scattering after an additional ultrasonic treatment within the measurement cell, which then revealed a bidisperse size distribution with a nanoparticle and a microparticle fraction. A higher chitosan ratio mainly influenced the nanoparticle fraction, which is attributed to the formation of additional, and slightly bigger pure chitosan nanoparticles during spray drying. Generally, due to the higher PLGA content about seven PLGA nanoparticles with a mean diameter of 519.7 nm were found in one chitosan microparticle with a mean diameter of 6.61 µm for low chitosan ratio, whereas, high chitosan ratio during spray drying resulted in an encapsulation of about two 519.7 nm-PLGA-nanoparticles per chitosan microparticle with a mean diameter of 5.75 µm. The achieved encapsulation efficiency of NiMPs was either 39 ± 19% (1/3 chitosan:2/3 PLGA) or 51 ± 16% (2/3 chitosan:1/3 PLGA); and therefore, not significantly different. Although, 1/3 more chitosan as matrix material was present, the encapsulation efficiency did not significantly increase. This is again attributed to the formation of additional pure chitosan nanoparticles, which were determined via static light scattering measurements after additional ultrasonic treatment. Consequently, spray drying is a very efficient method to encapsulate nanoparticles up to a mass ratio of 2/3, covering them successfully with the added matrix polymer, even more beneficial when using less matrix material. The polymer ratio variation had no impact on the following *ex vivo* experiments.

Mucosal uptake of PLGA nanoparticles and NiMPs was investigated, with porcine olfactory mucosa specimens, in specialized side-by-side cells, mimicking the upside-down orientation and physiological conditions within the nasal cavity at 35°C and 90% humidity. Overall, a size- and time-dependent uptake of PLGA nanoparticles was observed. Smaller PLGA nanoparticles, with 80 nm diameter, were taken up immediately deep into the *lamina propria* 5 min after application to the epithelium. Fewer 175 nm particles penetrated the epithelial and the basal layer within 5 min. For 520 nm PLGA nanoparticles, slower uptake was observed, reaching the outer ∼150 µm of the *lamina propria* within 10 min. Additionally, 80 and 175 nm particles were documented to directly associate with cell nuclei within 5 min, which implies intracellular uptake. In contrast, 520 nm particles have not initially been observed near nuclei, but after 15 min, and to lower amounts, compared to the smaller nanoparticles. The occurring decelerated penetration due to increasing particle size is already reported in the literature (Mistry et al., 2009; Bourganis et al., 2018). Further, reported critical particle size enabling transcellular transport after intranasal administration is 200 nm (Bourganis et al., 2018; Rabiee et al., 2020). However, Musumeci et al. (2018) reported that PLGA nanoparticles, up to 300 nm, reach and accumulate in the brain of rats, but did not clarify the occurring transport mechanism (Musumeci et al., 2018). Nanoparticles with diameters greater than 500 nm have not yet been reported for nose-to-brain (N2B) targeting (Bourganis et al., 2018; Rabiee et al., 2020), but size-dependent intracellular uptake is possible also for 200, 500 and even 1,000 nm particles in epithelial cells (Caco-2) (Win and Feng 2005).

Remarkably, we reported herein co-localization of 520 nm PLGA particles with nuclei, especially accumulated in neuronal bundles, which implies intracellular uptake in neuronal axons and could enable transcellular transport within the olfactory, or trigeminal nerve pathway to reach the brain and the central nervous system (CNS) (Gänger and Schindowski 2018; Bourganis et al., 2018; Rabiee et al., 2020; Keller et al., 2021). The diameter of axons in humans ranges from 100 to 700 nm (Morrison and Costanzo 1990; Morrison and Costanzo 1992; Mistry et al., 2009). It is broadly reported that directed transport along with neuronal cells and posterior neuronal bundles is one of the most promising routes for nose-to-brain delivery. To further investigate this phenomenon, additional experiments visualizing the exact cellular uptake and transport mechanism could be helpful. Hence, live imaging or *in vivo* experiments with particle sizes above 200 nm should again also be taken into account.

Contrasting the observations of 520 nm pure PLGA particles to the similar 520 nm PLGA particles embedded into chitosan, NiMPs were taken up faster and deeper in the *lamina propri*a. A quantitative analysis was herein not possible because sample sizes differed between PLGA nanoparticle suspension and NiMP powder samples. The effect of applied polymer amount and sample condition needs hence to be investigated in future studies. Within 15 min high amounts of NiMPs were detected spread over the whole olfactory mucosa section, reaching the collagen tissue at the basolateral side, where none of the purely applied PLGA nanoparticles were found. Furthermore, chitosan has been detected associated with the penetrated PLGA nanoparticles within the olfactory mucosa. Especially after 2 h, several 520 nm particles have been found together with chitosan in the outer zone of lymphoid follicles, where usually T-cells and B-cells are present in the porcine olfactory mucosa (Ladel et al., 2018). Small pure PLGA nanoparticles (80 and 175 nm) have also been detected in this area after approximately 5 min. In contrast to this, purely applied 520 nm PLGA particles were not found associated with lymphoid follicles. Ladel et al. (2018) showed CD3^+^, CD20^+^, and CD14^+^ cells in the outer area of the porcine olfactory epithelium-associated lymphoid follicles (Ladel et al., 2018). This finding further matches the subepithelial dome more prominent in intestinal Peyer’s patch but also found in young adults and sheep.

Also within this study, we consequently examined the general initial immune reaction, which is mediated by CD14^+^ immune cells, namely monocytes and macrophages. It takes place within the first few hours after contact with a biomaterial (Anderson et al., 2008). We further monitored the presence of CD14 around lymphoid follicles. In our study, PLGA was not found co-localized with CD14^+^ cells, thus seems not to be recognized by CD14. Therefore, it is assumed that PLGA nanoparticles can pass the olfactory epithelium reaching for example, neuronal bundles, and could then be further transported to the brain and the CNS. Nevertheless, 80 nm PLGA nanoparticles especially have been documented associated with lymphoid follicles, which should be further clarified. Although this result is not attributable to the recognition by monocytes or macrophages, an interaction mechanism with other present immune cells could still be possible and should be explored in future studies.

The chitosan penetration gradient within the olfactory mucosa was found in our study to be proportional to a gradient of the initial immune response. Chitosan, therefore, seemed to cause a migration of CD14^+^ cells to the particles underlying the *lamina propria* within 15 min after particle application to the epithelium. The membrane located glycoprotein CD14 forms together with the lipopolysaccharide-binding protein (LPB), the lipopolysaccharide-receptor (LPR), which recognizes bacterial endotoxin lipopolysaccharides lipopolysaccharide-receptor present on the surface of Gram-negative bacteria (Wright et al., 1990). Lipopolysaccharide-induced upregulation of the CD14 membrane receptor is known as an initial immune reaction in mucosal tissues, such as the intestine (Funda et al., 2001); (Frolova et al., 2008). Otterlei et al. (1994) initially described chitosan-recognition by CD14 on monocytes (Otterlei et al., 1994). To the contrary, Qiao et al. (2010) reported the reduction of pro-inflammatory cytokines through chitosan oligosaccharides (Qiao et al., 2010). Recently, Chang et al. (2019) differentiated between low molecular (below 7.1 kDa) and high molecular (above 72 kDa) chitosan in the macrophage-binding mechanism. They reported that only low molecular weight chitosan up to 7.1 kDa would bind CD14 and interpreted it as an activation of a pro-inflammatory cascade. Studies describing the immune reaction of applied chitosan microparticles are still rather poor. Solely, S. Davis et al. (2018) proved chitosan particles with a high degree of deacetylation with sizes of 1–10 µm causing less M1-macrophage activation than similar chitin particles (Davis et al., 2018). Consequently, either the pro- or anti-inflammatory ability of chitosan particles is dependent on molecular weight, particle size, and degree of deacetylation. Taking this into account, we assume that in our study the observed CD14^+^ signal should be anti-inflammatory inducing a wound healing cascade, because the herein utilized chitosan has a molecular weight of 150–300 kDa. This should hence not be critical because Chang et al. (2019) reported for chitosans with molecular weights above 72 kDa solely anti-inflammatory reactions. Further, a high degree of 80% deacetylation and lastly a particle size ranging through agglomerates from approximately one micron up to 100 µm supports this reasoning. Nevertheless, the herein investigated CD14 immunohistochemical staining does not allow us to distinguish between M1- and M2-macrophages, which should be further evaluated in cell culture experiments, because, for example, a quantitative cell assay with altering cytokine concentrations would be needed as a reference (Zarif et al., 2016). This was not possible with the *ex vivo* samples of this study.

Moreover, the chitosan coating of NiMPs revealed several other interesting findings different from the purely applied identical PLGA nanoparticles. Chitosan was found to penetrate the olfactory mucosa approximately ∼150 µm within 15 min. Further, it accelerated the penetration of coated nanoparticles significantly and enables transport over the whole transverse tissue section within 15 min as described above. This is attributed to the swelling of chitosan on the top of the mucosa because the naturally occurring pH value in the nose of healthy individuals ranges around pH 6 (England et al., 1999). Unmodified chitosan does not completely dissolve at physiological pH values in the nose due to its pKa value of ∼6.5 but it is swelling and single chitosan polymer chains can move into the tissue. One explanation of enhanced uptake and accelerated permeation into the olfactory mucosa could be attributed to the well-known ability of chitosan to open tight junctions or more general cell-cell junctions enabling paracellular transport. The membrane protein *Zonula occludens* protein 1 (*ZO-1*) is part of tight junctions within the epithelial cell layer as well as endothelial cells, and adherens junctions within the *lamina propria* (Steinke et al., 2008; Wolburg et al., 2008). When tight junctions are opened due to chitosan treatment, the membrane protein *ZO-1* immunohistological staining fades. V. Dodane et al. previously reported this in 1999 in the cell culture of Caco-2 cells. The underlying mechanism caused by the internalization of this specific protein from the membrane to the cytoskeleton was later clarified by Smith et al. (2004) (Smith et al., 2004). This ability in cell culture has been proved by several research groups in the past decades (Dodane et al., 1999; Rassu et al., 2016); nevertheless, the chitosan application onto tissues is rarely reported. The opening mechanism in epithelial cells, previously reported by Dodane et al. (1999) to take place after 1 h and regenerate within 24 h also observed through fading and recovery of *ZO-1* staining (Dodane et al., 1999), however, they investigated acidic solutions at low chitosan concentrations. Smith et al., 2004 further described a concentration-dependent internalization of *ZO-1* on Caco2-monolayers within 1 h. M. Fazil et al. (2012) additionally showed increased brain uptake caused by chitosan particles but did not show *ZO-1* staining as reference (Fazil et al., 2012). We, herein, showed for the first time that this is also true in the naturally occurring environment of porcine olfactory mucosa as a prominent biological barrier. The *ZO-1* discoloring is attributed to internalization and opening of cell-cell junctions occuring in the *ex vivo* setting within only 15 min, including swelling of the dry chitosan-coated particles, which was never previously reported. To better understand which dosage of chitosan is most effective to promote paracellular transport, further experiments with different concentrations, particle amounts, as well as shorter timeframes would be interesting.

Finally, local particle clearance was observed above surface-located bowman glands. NiMPs have been cleared completely within 30 min, which could be attributed either to accelerated particle uptake within glands or ongoing mucus production resulting in continuously pushing away the overlaying particle layer. Ongoing mucus production even in *ex vivo* setting was already early described to last at least 4–5 h for frog palate mucosa (Sadé et al., 1970; Aspden et al., 1995), later also for human specimens (Aspden et al., 1997) and ferret trachea with velocities up to 9.5 ± 3.5 mm min^−1^ (Abanses et al., 2009; Jeong et al., 2014). Further, chitosan was found to reduce mucociliary transport rate (MTR) proportionally to its molecular weight (Aspden et al., 1995), which was attributed only to its physical interaction with mucins not harming the mucosa itself (Aspden et al., 1997). Unfortunately, no further tracking of particles was possible in this study because no particles have been detected directly associated with the gland ensheathing cells, or the underlying cells of the *lamina propria,* and continuous monitoring was not possible due to the sampling procedure. As Raber et al. (2014) also described enhanced uptake of chitosan-coated PLGA nanoparticles in glandular ducts of hair follicles (Raber et al., 2014), mucosal glands could also function as uptake enhancing mucosa components. For the clarification of the role of surface-associated glands, it would be interesting to continuously monitor the site of action. It would be beneficial to monitor organoids, such as glands, using a complex model of the olfactory mucosa. For example an organ-on-a-chip model, a so-called nose-on-a-chip.

## Conclusion

Within this study, we successfully developed specialized Nano-in-Micro particles (NiMPs) consisting of PLGA nanoparticles embedded into chitosan microparticles via spray drying. We evolved a robust spray-drying process resulting in homogenous microparticles with high encapsulation efficiency, uniform size distribution, smooth surface, and dense pores. We demonstrated, that not only 80 and 175 nm PLGA nanoparticles purely applied to the olfactory mucosa can be taken up intracellularly within only 5 min, but also 520 nm PLGA particles are associated with nuclei and neuronal fibers after 15 min, which can imply transcellular transport within the olfactory epithelium and intracellular uptake into neuronal cells following transport along those to the olfactory or trigeminal nerve, which finally enables targeting of the brain and the central nervous system (CNS). These findings were further proved to be size-time-proportional, resulting in smaller particles moving faster (within only 5 min) and to a higher extent into the *lamina propria*. Chitosan-coated NiMPs were identified as even more interesting nanoparticle carriers and are auspicious delivery vehicles themselves. Their chitosan coating is subsequently swelling on the mucosal barrier; therefore, single chitosan polymer chains penetrate within 15 min approximately 150 µm into the olfactory mucosa. With this, the chitosan penetration affects the opening of tight junctions and, therefore, can additionally enable paracellular transport through the olfactory epithelium. Consequently, the accelerated uptake of chitosan-coated nanoparticles was examined.

PLGA nanoparticles, as well as, NiMPs were found in lymphoid follicles; however, only NiMPs were co-localized after 15 min with the immunoreactivity signal of membrane protein CD14 present on monocytes and macrophages. Hence, we assume any other interaction for PLGA nanoparticles e.g., T- or B-cell mediated binding, which needs to be investigated in future studies. NiMPs herein showed the ability to initialize an anti-inflammatory cascade within 15 min after particle application, due to the beneficial properties of the herein utilized chitosan (molecular weight, degree of deacetylation, and particle size). However, CD14^+^ macrophages could be either pro-inflammatory M1-macrophages or anti-inflammatory M2-macrophages effecting wound healing, which could not be fully clarified within this study because therefore cytokine levels are needed as a reference, which should be investigated in cell culture. Additionally, particle clearance of gland overlaying particles was herein documented, but could not be fully enlightened, because the sampling procedure was limited and continuously monitoring was not possible.

In conclusion, within this study, we demonstrated the possibility for PLGA nanoparticles as well as for chitosan NiMPs to take all three prominent pathways to the brain and the CNS, namely transcellular, intracellular via neuronal cells, and paracellular transport. Overall, although polymeric particles are broadly reported as promising drug delivery systems, not only for intranasal application the usage in the clinic is still lacking. Up to now, no nano-based system has entered advanced clinical trials. Consequently, still more research is needed to collect additional data and unravel the complex phenomena within the nasal mucosa, to clarify the different transport mechanisms and the role of special organoids, like bowman glands. Further, verifying specific influences of particle material, other formulation components, and particle sizes.

## Data Availability

The original contributions presented in the study are included in the article/Supplementary Material, further inquiries can be directed to the corresponding author.
