# Supplementary material for: Nano-in-Micro-Particles Consisting of PLGA Nanoparticles Embedded in Chitosan Microparticles via Spray-Drying Enhances Their Uptake in the Olfactory Mucosa
Source: Front Pharmacol. 2021 Sep 1;12:732954. doi: 10.3389/fphar.2021.732954 (PMC8440808; doi:10.3389/fphar.2021.732954)
Supplement: Supplementary file 1 [file DataSheet1.docx]

Supplementary Material

# Supplementary Tables

Supplementary Table 1. Lasers and corresponding emission filters utilized for Confocal Laser Scanning Microscopy (CLSM) of different fluorophores with a confocal LSM 710 (Carl Zeiss Microscopy Deutschland GmbH, Oberkochen, Germany).

| **Fluorophore** | **Laser** | **Emission filter** |
| --- | --- | --- |
| DAPI (4′,6-Diamidin-2-phenylindol 2HCl) | 405 nm | 410-501 nm |
| Fluorescein-5-isothiocyanat (FITC) | 488 nm | 493-562 nm |
| Fluorescein sodium salt | 488 nm | 493-562 nm |
| Lumogen | 514 nm | 566-703 nm |
| Alexa Fluor® 647 | 633 nm | 638-755 nm |

# Supplementary Figures


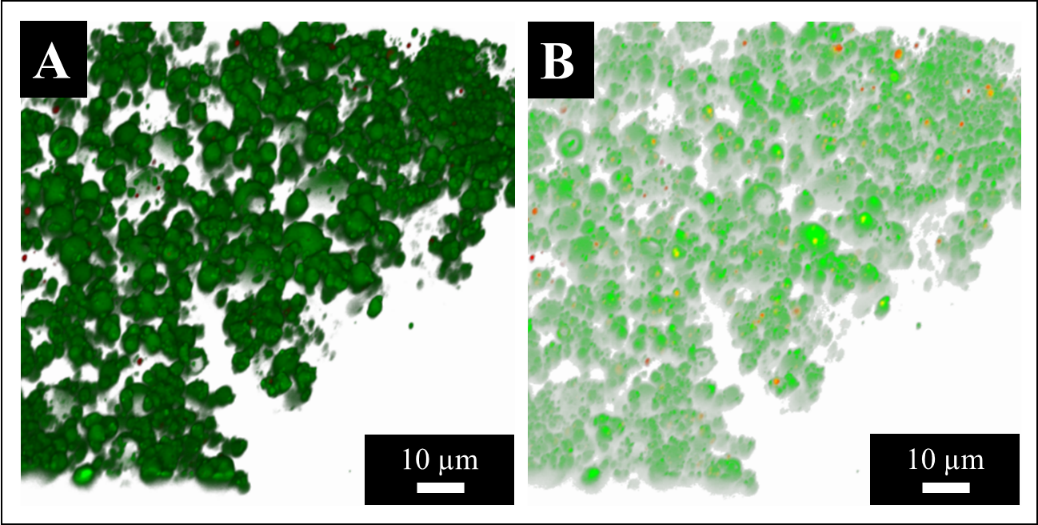


Supplementary Figure 1. Spray dried nano-in-micro particles (NiMPs) consisting of 1/3 PLGA nanoparticles (520 nm) and 2/3 chitosan by weight. A: 3D projection of Confocal Laser Scanning Microscopy (CLSM) images (green: chitosan; red: PLGA) stiched together from a z-stack, mag. 630 X, external view. B: Transparent 3D projection of CLSM images (green: chitosan; red: PLGA) stiched together from a z-stack, mag. 630 X, internal view.

Supplementary Figure 2. Thickness of porcine olfactory mucosa explants after permeation experiment fixed and stained with Hematoxylin-Eosin (HE) staining. Thickness differs not significantly between individual samples (n=3). Statistical analysis was performed with Shaipiro-Wilk normality test, One-Way-ANOVA with Levene’s variance test and Bonferoni posthoc test at a significance niveau of α=0.05 respectively.


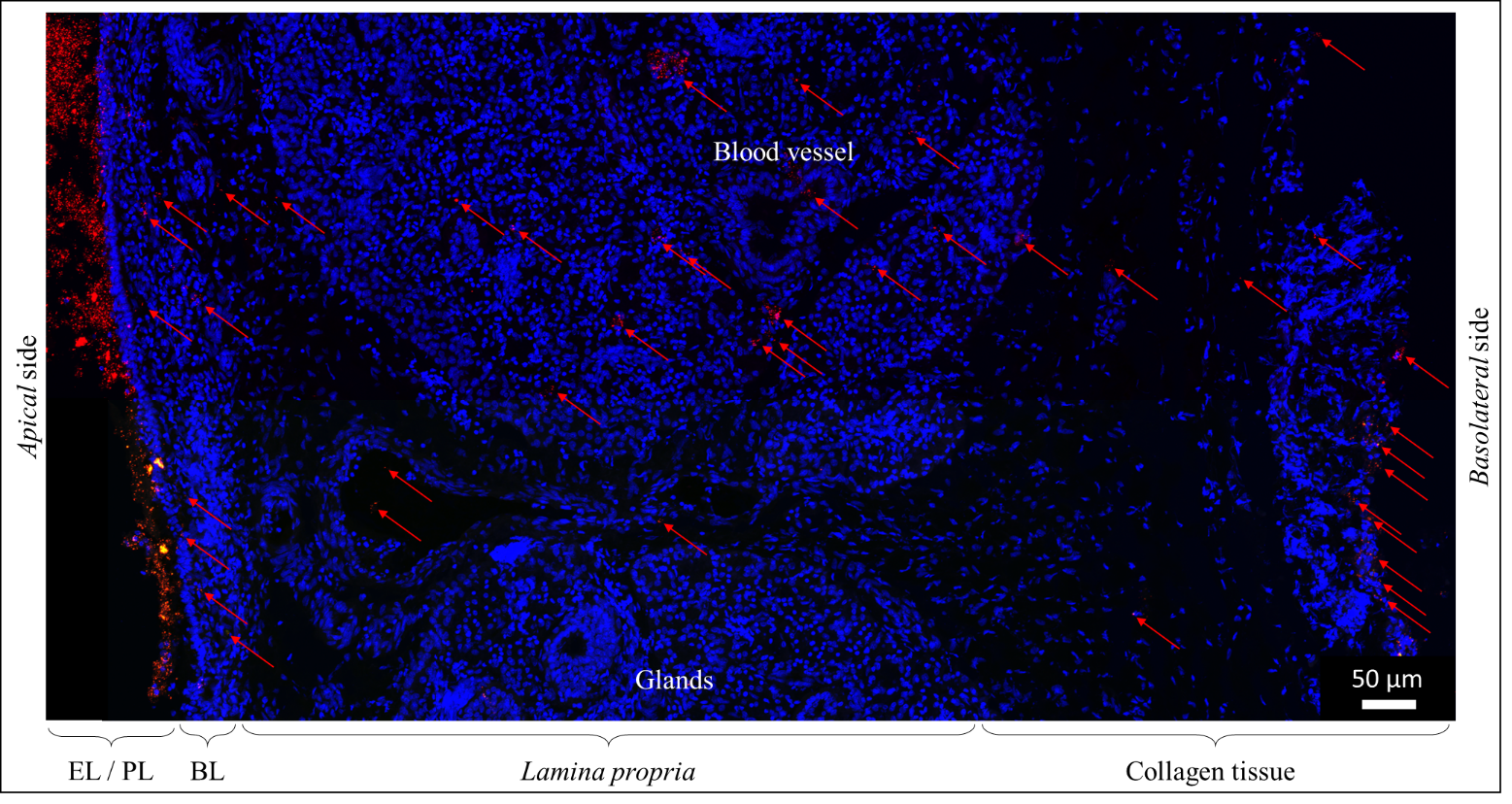


Supplementary Figure 3. Permeation of nano-in-micro particles (NiMPs) consisting of PLGA nanoparticles (520 nm; red) encapsulated in chitosan applied to the apical side of porcine olfactory mucosa. Confocal Laser Scanning Microscopy (CLSM) images stitched together from a z-stack. Cell nuclei stained with DAPI (blue). Red arrows mark particles. Chitosan labeled with fluorescein (green). Overlapping signals of red (nanoparticles) and green (chitosan) appear yellow. Image taken 15 min after application. EL: Epithelial cell layer; PL: Particle layer; BL: Basal cell layer.


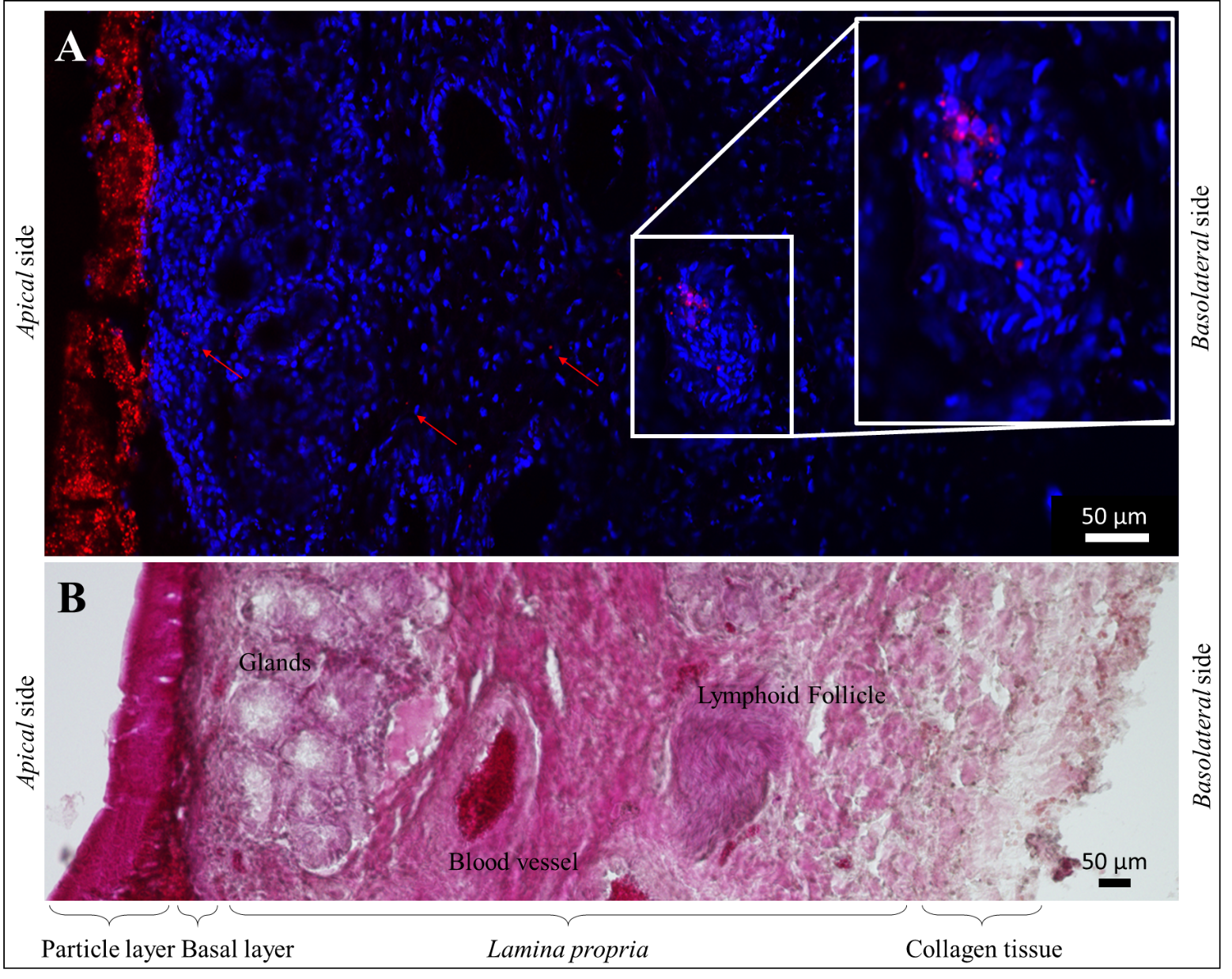


Supplementary Figure 4. Permeation of nano-in-micro particles (NiMPs) consisting of PLGA nanoparticles (520 nm; red) encapsulated in chitosan. Images taken 15 min after application. A: Confocal Laser Scanning Microscopy (CLSM) image stitched together from a z-stack; Cell nuclei stained with DAPI (blue), red arrows mark single particles. B: Light Microscopy (LM) image; Hematoxylin-Eosin (HE) staining.


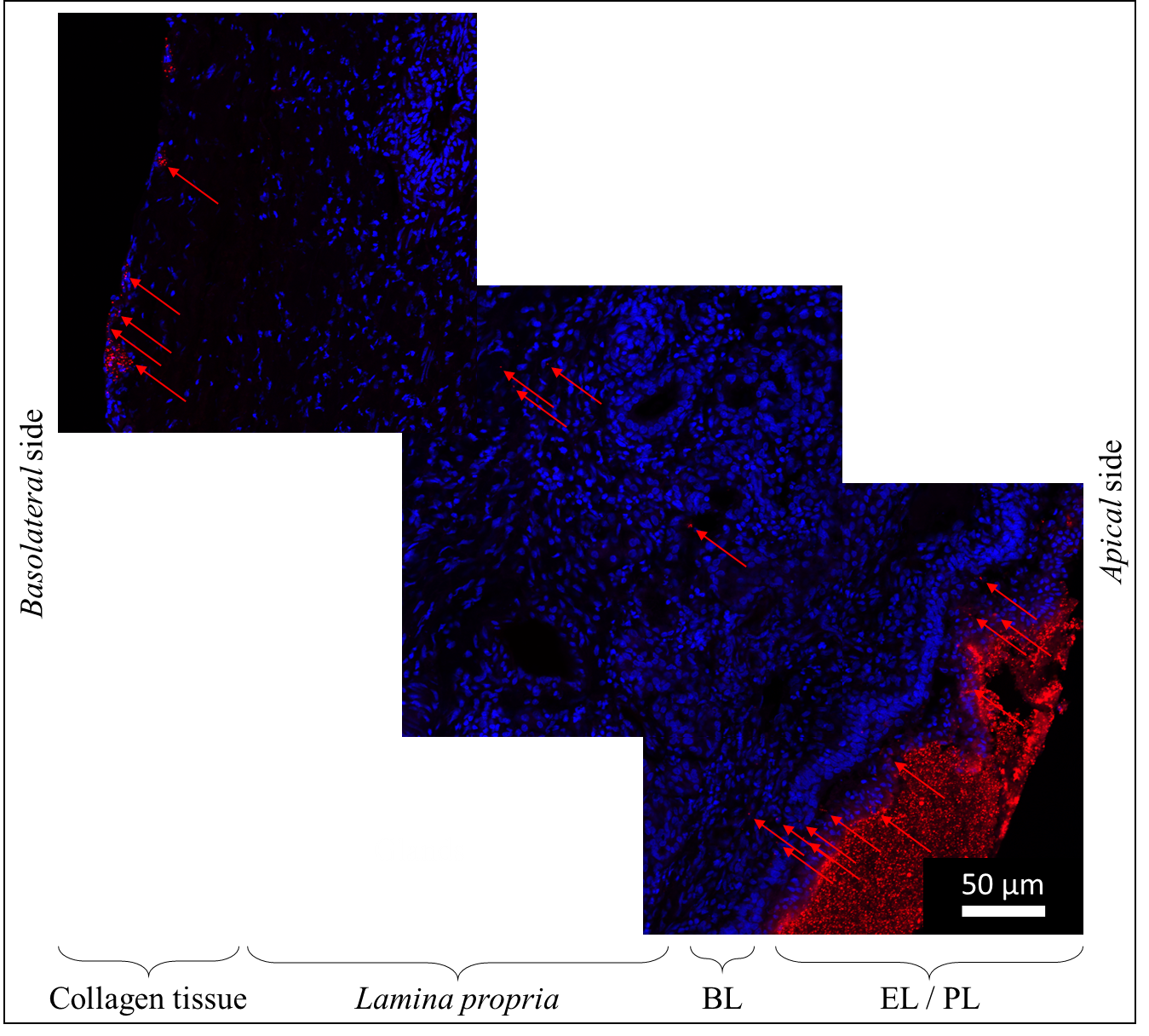


Supplementary Figure 5. Permeation of nano-in-micro particles (NiMPs) consisting of PLGA nanoparticles (520 nm; red) encapsulated in chitosan applied to the apical side of porcine olfactory mucosa. Confocal Laser Scanning Microscopy (CLSM) images stitched together from a z-stack. Cell nuclei stained with DAPI (blue). Red arrows mark particles; Chitosan unlabeled; Image taken 2 h after application. EL: Epithelial cell layer; PL: Particle layer; BL: Basal cell layer.


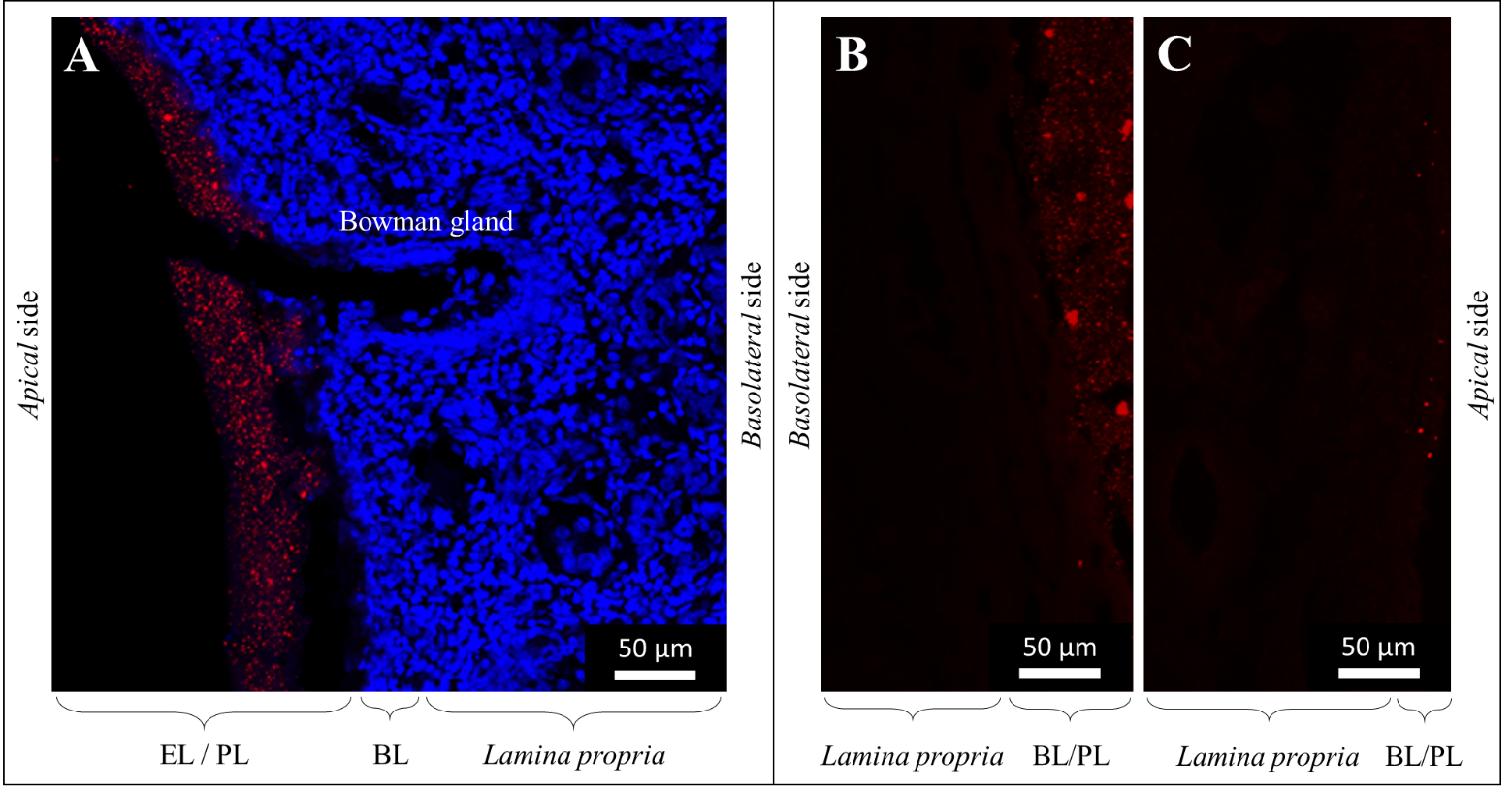


Supplementary Figure 6. Permeation of nano-in-micro particles (NiMPs) consisting of PLGA nanoparticles (520 nm; red) encapsulated in chitosan applied to the apical side of porcine olfactory mucosa. Confocal Laser Scanning Microscopy (CLSM) images. A: Cell nuclei stained with DAPI (blue). Chitosan unlabeled, image taken 30 min after application. B: NiMPs 15 min after application to the apical side, PLGA nanoparticles (red) belonging to Figure 4F original manuscript. C: PLGA nanoparticles (red) 15 min after application to the apical side belonging to Figure 4A original manuscript. EL: Epithelial cell layer; PL: Particle layer; BL: Basal cell layer.
